# CIRI: an efficient and unbiased algorithm for *de novo* circular RNA identification

**DOI:** 10.1186/s13059-014-0571-3

**Published:** 2015-01-13

**Authors:** Yuan Gao, Jinfeng Wang, Fangqing Zhao

**Affiliations:** Computational Genomics Lab, Beijing Institutes of Life Science, Chinese Academy of Sciences, Beijing, 100101 China; University of Chinese Academy of Sciences, Beijing, 100049 China

## Abstract

Recent studies reveal that circular RNAs (circRNAs) are a novel class of abundant, stable and ubiquitous noncoding RNA molecules in animals. Comprehensive detection of circRNAs from high-throughput transcriptome data is an initial and crucial step to study their biogenesis and function. Here, we present a novel chiastic clipping signal-based algorithm, CIRI, to unbiasedly and accurately detect circRNAs from transcriptome data by employing multiple filtration strategies. By applying CIRI to ENCODE RNA-seq data, we for the first time identify and experimentally validate the prevalence of intronic/intergenic circRNAs as well as fragments specific to them in the human transcriptome.

**Electronic supplementary material:**

The online version of this article (doi:10.1186/s13059-014-0571-3) contains supplementary material, which is available to authorized users.

## Background

The past 20 years have witnessed much progress in the study of RNAs [1,2]. A large proportion of known RNAs were proved to undertake diverse important biological functions. Circular RNA (circRNA), one of the latest star RNAs, is an RNA molecule with ends covalently linked in a circle that has been discovered in all domains of life with distinct sizes and sources [3-8]. While in eukaryotes circRNAs were often regarded as transcriptional noise, such as products of mis-splicing events [9], recent studies using high-throughput RNA-seq data analysis and corresponding experimental validation have proved that they actually represent a class of abundant, stable and ubiquitous RNAs in animals [10-13]. Their high abundance and evolutionary conservation between species suggest important functions, and studies subsequently revealed that a subset of them function as microRNA sponges [11,14]. Nonetheless, the functions of the majority of circRNAs still remain unknown and there are few models of their mechanism of formation, which prevents model-oriented experimental validation to solve the circRNA mystery.

Our ignorance about circRNAs is partly due to an insufficiency of sequencing data specifically aimed at circRNA detection. In contrast to the scarcity of these data sets, large amounts of RNA-seq data have been generated using high throughput sequencing technology. Analyzing circRNAs identified from enormous RNA-seq data combined with sequencing data generated from additional samples has been adopted in several studies [11,13] and will probably continue to be a commonly used approach in further studies on circRNAs. Thus, an all-round computational tool for unbiased identification of circRNAs from various RNA-seq data sets becomes necessary. Development of such a detection tool, however, is difficult due to the non-uniformity of RNA-seq data sets and the complex nature of eukaryotic transcription: (i) a large proportion of circRNAs have relatively low abundance compared with their linear counterparts [10,15], while most RNA-seq data were generated without a circRNA enrichment step, such as RNase R treatment, which makes it difficult to accurately distinguish circRNAs from false positives caused by noise in RNA-seq data; (ii) existing annotations of reference genomes were mainly based on linear RNA transcript analyses, which is not applicable for circRNA identification, and non-model organisms often have incomplete gene annotation or even lack gene annotation; (iii) read lengths vary in different sequencing data sets, which challenges unbiased identification of circRNAs; (iv) complexities of eukaryotic transcription may generate other non-canonical transcripts, such as lariats and fusion genes, in which corresponding reads similar to circular junctions may lead to false discoveries.

Therefore, current algorithms for circRNA detection have been mainly developed for certain data sets, which restricts their utility as a universal approach. In 2012, Salzman *et al*. [13] proposed an annotation-dependent algorithm in which circRNAs were detected based on the alignment of reads to a customized database of annotated exon boundaries. They also improved the algorithm in a more recent report by adding false discovery rate (FDR)-controlled filtration based on statistics of alignment quality scores [10]. Nevertheless, their approach necessitates annotation and is not, therefore, applicable to species that are incompletely annotated. Besides, the filtration based on statistics may not be effective on low coverage regions or most RNA-seq data that is not sequenced deeply enough. Memczak *et al.* [11] utilized GT-AG splicing signals flanking exons as a filter for *de novo* identification of circRNAs; most recently, a similar pipeline was used to search for microRNA-sponge candidate circRNAs [16]. However, both algorithms adopt a two-segment alignment of split reads, which may lead to an inability to detect certain types of circRNAs with more complicated alignments (for example, short exon-flanking circRNAs). Moreover, the filtration strategy employed in these algorithms is insufficient for removal of false positives. Jeck *et al*. [12] adopted another strategy, which compares untreated and RNase-treated sequencing results to confirm the existence of circRNA candidates and remove false positives. This approach is sensitive and able to estimate the relative abundance of circRNAs. However, it may introduce systematic bias in the enrichment procedure, and also is not applicable to the majority of currently available RNA-seq data generated without circRNA enrichment.

Compared with circRNA detection algorithms, mapping algorithms have a much longer history of development, and some of them were specifically designed for split and local alignment. BWA-MEM [17] implements a local alignment by seeding with maximal exact matches and extension with an affine-gap algorithm, which provides for fast speed and high accuracy. Another algorithm, segemehl [18], uses an enhanced suffix array for seeding and was reported to outperform its competitors on splice site detection. Because circRNAs are characterized by circular junctions, which resemble splicing and usually produce multiple alignments during read mapping, these mature mapping algorithms may provide large improvements in accuracy and efficiency and may make unbiased detection of circRNAs possible. Indeed, an auxiliary script in segemehl simply summarizes and reports junctions of circular candidates as well as splice sites. However, without strategies to identify sequential features peculiar to circRNAs, large amounts of false positives are unavoidable.

In this article, we present a comprehensive computational tool for circRNA identification and annotation from RNA-seq reads. In contrast to other annotation- or circRNA enrichment-dependent algorithms, this method employs a novel algorithm based on paired chiastic clipping (PCC) signal detection in the Sequence Alignment/Map (SAM) of BWA-MEM combined with systematic filtering to remove false positives. Application of our algorithm to existing and newly generated sequencing data in this study combined with experimental validation demonstrate its reliability and potential for further studies on circRNAs.

## Results

**Figure 1:**
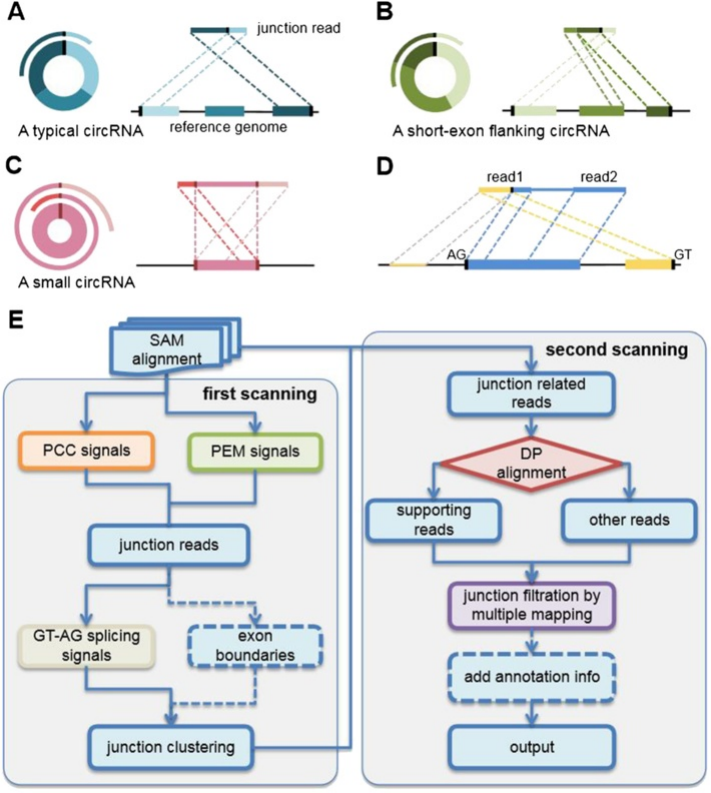
**The basis and pipeline of circRNA identification in CIRI. (A-C)** CIGAR signals identified in the first scan. **(A)** For most circRNAs, two segments of junction reads align to the reference sequence separately in reverse orientation. **(B)** If one segment is longer than the exon flanking junction, the rest of the segment can align to the nearby exon contained in the circRNA. **(C)** If the circRNA length is shorter than the read length, two terminal segments will possibly align to the termini of the area where the middle segment aligns. **(D)** To reduce the false positive rate, candidate circRNAs are filtered based on the following information: i) PEM signals - the paired read of a junction read should align within the inferred circRNA area; ii) GT-AG splicing signals should be present in the inferred junctions; iii) mapping statistics - mapping quantity and quality, and mapping read length in the junctions. **(E)** The CIRI pipeline for detecting circRNAs from transcriptome data. (DP: dynamic programming).

Our circRNA identification tool, named 'CIRI' (CircRNA Identifier), scans SAM files twice and collects sufficient information to identify and characterize circRNAs (Figure 1). Briefly, during the first scanning of SAM alignment, CIRI detects junction reads with PCC signals that reflect a circRNA candidate. Preliminary filtering is implemented using paired-end mapping (PEM) and GT-AG splicing signals for the junctions. After clustering junction reads and recording each circRNA candidate, CIRI scans the SAM alignment again to detect additional junction reads and meanwhile performs further filtering to eliminate false positive candidates resulting from incorrectly mapped reads of homologous genes or repetitive sequences. Finally, identified circRNAs are output with annotation information. Details of the CIRI algorithm are provided in Materials and methods.

### Simulation studies

Since neither a large database of validated circRNAs nor a specific simulation tool for the non-canonical transcripts is available to date, a simulation tool (CIRI-simulator) developed by us was used to generate simulated reads and evaluate the performance of CIRI. We first focused on how expression levels of circRNAs could affect the performance of CIRI. As shown in Figure S1A in Additional file 1, with a simulated increase in coverage of circular transcripts from 3- to 10-fold, the sensitivity of circRNA identification rose steadily, and CIRI maintains high sensitivity of 93% for circRNAs at a depth of 10-fold or higher. Notably, even at the low depth of three-fold, more than 70% of circRNAs could be detected by CIRI. We also tested the performance of CIRI on variable read length. As shown in Figure S1B in Additional file 1, for most commonly generated read lengths in Illumina platforms to date, CIRI shows high sensitivity and low FDR. These results indicate that CIRI is most efficient for reads with lengths ranging from 60 to 100 bp, and it can also be used for shorter reads, such as 40 bp reads, with a relatively low sensitivity. circRNAs of different sizes may have different types of PCC signals (described in Materials and methods) and this likely affects the performance of CIRI. We thus specifically explored the detection results of CIRI for a simulated data set (80 bp paired-end reads; depth of 10-fold for circRNAs). Simulated circRNAs were divided into four subsets according to their size relative to the read length: smaller than the read length (≤80 bp), twice the read length (81 to 160 bp), three-fold the read length (161 to 240 bp) and more than three-fold the read length (>240 bp). CIRI detection showed a slightly fluctuating trend for sensitivity, and for each of the four subsets, CIRI could identify more than 90% of the simulated circRNAs (Figure S1C in Additional file 1). Comparisons were performed between CIRI and segemehl, the latter of which was reported as a mapping algorithm applicable to circRNA reads [18]. With no annotation provided in the comparisons, CIRI showed good performance for all simulated data with different read lengths and sequencing depths (Figure S2 in Additional file 1).

Considering eukaryotic transcription complexities that may affect detection of circRNAs, the FDR in the above simulation studies may be underestimated. Therefore, we further utilized real data sets of poly-A selected sequencing as a blank background for our simulations. circRNAs have no poly(A) tails and could theoretically escape from sequencing based on poly-A purification, so poly-A selected sequencing data sets that contain complex information about the transcriptome can be ideal blank backgrounds and help us accurately estimate FDRs resulting from use of CIRI. We selected three poly-A selected sequencing data sets with different read lengths (54 bp, 76 bp, 101 bp) generated by three independent laboratories and added simulated reads of 10-fold circRNAs with corresponding read lengths to each. As shown in Figure S3A in Additional file 1, with no annotation provided, CIRI shows sensitivity consistent with the simulation tests on read length described above and could simultaneously control FDR at a low level. To better understand the FDR of CIRI, we also made further comparisons between CIRI and segemehl on the three data sets. While segemehl could detect similar numbers of simulated circRNAs, it showed FDRs approximately 20 times higher than CIRI for all three data sets when both used default settings (Figure S3A in Additional file 1). Other parameter settings recommended by segemehl were also tested, but FDR was reduced at the cost of sensitivity (Figure S3B,C in Additional file 1). As segemehl cannot utilize PEM information, we also applied the SE mode of CIRI to figure out if the discrepancy in performance could be totally attributed to the PEM filtering that CIRI employed. The results showed that although the lack of PEM information resulted in the FDR more than doubling, CIRI still showed better performance with regard to both sensitivity and FDR compared with the optimal setting of segemehl (Figure S3D in Additional file 1), which indicates the high efficiency of the filtering strategies employed by CIRI.

### Circular RNA validation based on sequencing of RNase R free/treated samples

**Figure 2:**
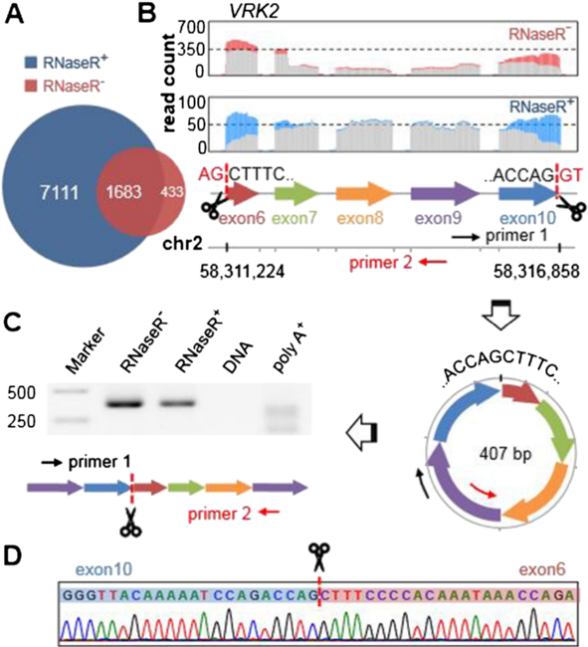
**Circular RNA validation based on sequencing of RNase R treated/untreated samples and details of circRNA chr2: 58,311,224|58,316,858. (A)** Overlap of prediction results between two samples (RNaseR^+^, ribominus RNA treated with RNase R; RNaseR^-^, ribominus RNA). **(B)** Coverage of five exons contained in chr2: 58,311,224|58,316,858 in the two samples (red, junction reads identified by CIRI in RNaseR^-^ sample; blue, junction reads identified by CIRI in RNaseR^+^ sample; grey, other reads). Scissors indicate the splicing sites, which are flanked by GT-AG signals highlighted in red. **(C)** circRNA structure and its linear amplified fragment using a pair of outward-facing primers. **(D)** Sequencing chromatogram of the PCR product across the junction.

To verify that CIRI identified *bona fide* circRNAs rather than false positives, we generated 7.4 Gb and 16.3 Gb sequence data from HeLa cells based on ribominus RNA sequencing with or without RNase R treatment (RNaseR^+/-^, respectively). RNase R is a magnesium-dependent 3′ → 5′ exoribonuclease that digests essentially all linear RNAs but does not digest lariat or circular RNA structures. Both data sets were used for prediction of circRNAs. As is shown in Figure 2A, predictions by CIRI show a significant overlap between the two data sets. About 80% of candidate circRNAs from the RNaseR^-^ sample that have at least five supporting junction reads were also detected in the RNaseR^+^ sample.

**Figure 3:**
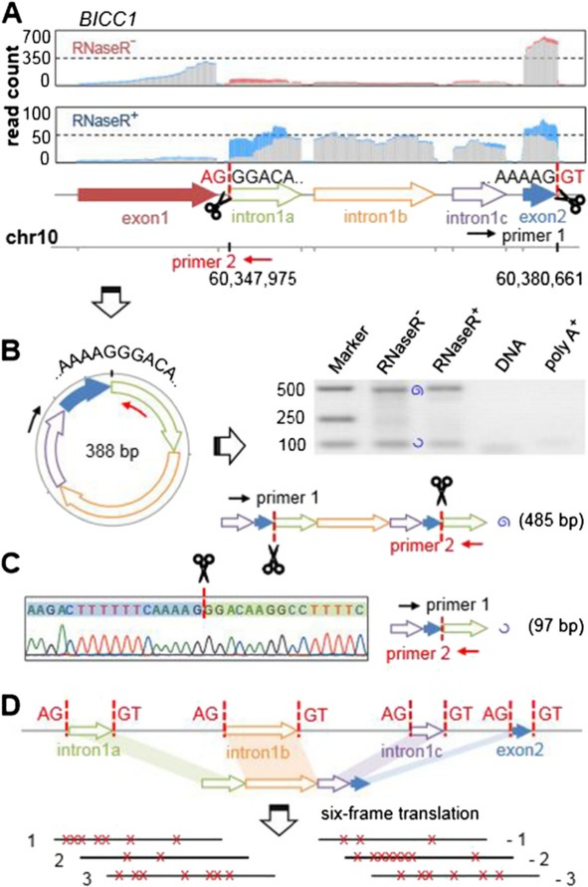
**A non-exonic circRNA with intronic/intergenic circRNA fragments. (A)** Coverage of one exon and three intronic regions contained in chr10: 60,347,975|60,380,661 in the two samples (red, junction reads identified by CIRI in RNaseR^-^; blue, junction reads identified by CIRI in RNaseR^+^; grey, other reads). **(B)** circRNA structure and its two linear amplified fragments using a pair of outward-facing primers. The top PCR product in the gel is longer than one complete circle around the circRNA and the bottom PCR product is shorter than one complete circle. **(C)** Sequencing chromatogram of the PCR product across the junction. **(D)** The circRNA contains three intronic circRNA fragments and one exon, which are all flanked by GT-AG splicing signals. The positions of stop codons in all six frames are shown as crosses.

We randomly selected 33 candidate circRNAs with relatively high expression levels (more than five junction reads) and designed outward-facing primers for each to amplify the fragment across the junction from cDNA synthesized from total RNA RNaseR^-^, RNaseR^+^ and poly-A^+^ samples. Considering residual genomic DNA in total RNA may generate false positive amplification and thus affect validation, we also implemented PCR using the same conditions with total DNA as a negative control. As shown in Table S1 in Additional file 1, we successfully amplified 24 circRNAs (73% of 33 candidates) from RNaseR^-^ and RNaseR^+^ samples. To distinguish from canonical mRNA transcripts, the coordinate positions for each circRNA are connected with a vertical bar '|' instead of a dash '-'. Here the vertical bar represents the junction of circRNAs. Because some candidate circRNAs contain extremely short exons or introns with exceptional GC content, which is quite challenging for primer design, our validation approach would underestimate the specificity of CIRI. Details for each validated circRNA are depicted in Figures 2 and 3 and Figures S4, S5 and S6 in Additional file 1. For all of the 24 validated circRNAs, no product with expected length could be amplified in the genomic DNA, which rules out the possibility of DNA contamination. We also found that although most validated circRNAs could not be amplified from poly-A selected RNA, weak PCR products do appear in a few cases. Since some circRNAs were detected from sequencing data of poly-A selected RNA in previous studies [19], these PCR products should result from residual circRNAs during poly-A selection.

All PCR products were further validated using Sanger sequencing and we also checked the RNA-seq mapping details (mapping quality, coverage and flanking splicing signals) for each of them. For example, amongst the five exons contained in chr2: 58,311,224|58,316,858 of gene *VRK2*, exon 6 is 50 bp long, which is much shorter than the read length (101 bp) and may result in related algorithms being unable to detect it (Figure 2; Figure S6A in Additional file 1). From the sequencing coverage plots for both the RNaseR^-^ and RNaseR^+^ samples, the high efficiency of CIRI detection of junction reads can be clearly observed, which demonstrates that CIRI can unbiasedly detect circRNAs with a short exon flanking the junction owing to its comprehensive consideration of multiple-segment style in junction read mapping (see Materials and methods).

The intronic circRNA chr10: 60,347,975|60,380,661 of gene *BICC1* is composed of four separated fragments in the human genome, and three of them are from the approximately 42 kb long intervening intron between exon 1 and exon 2 (Figure 3A; Figures S6B and S7 in Additional file 1). The three intronic circRNA fragments (ICFs) are 100 bp, 170 bp and 71 bp long, respectively, and they are all flanked by GT-AG splicing signals. It should be noted that this intronic circRNA is expressed at very low levels compared with its neighboring exons in the RNaseR^-^ sample. However, after treatment with RNase R, which nearly digested all the linear mRNA transcripts, we can clearly see the patterns of circRNA expression (Figure 3A-C). We translated the nucleotide sequence of this circRNA in all six possible frames, but found that none of them could generate a full length open reading frame (Figure 3D). This indicates that the three ICFs are unlikely to be novel exons. Furthermore, we checked currently available human gene expression databases, but found no evidence for their presence as linear transcripts, implying that they might be circRNA-specific sequences in the genome. Comparative genomic analysis revealed that these ICFs and their splicing signals are highly conserved in all currently available primate genomes (data not shown). Another non-exonic circRNA is chr5: 10,213,603|10,224,173, which is categorized as an intergenic circRNA by CIRI (Figure S5A in Additional file 1). Two ICFs, flanked by GT-AG splicing signals as well, act as splice donor and acceptor of the circular junction and form the whole circRNA. Indeed, we also found ICFs with similar features in all other three validated non-exonic circRNAs, which are distinct from the mapping pattern of lariats (Figures S4A-D and S8 in Additional file 1).

### A direct comparison between CIRI and other algorithms

**Figure 4:**
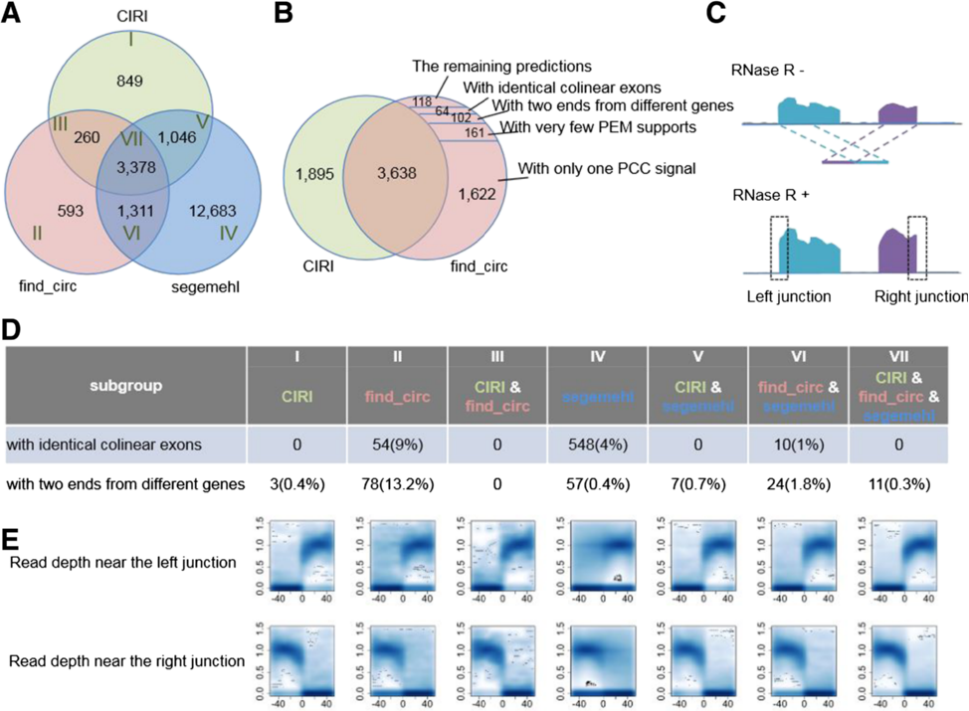
**Comparison between CIRI, find_circ and segemehl based on HeLa cell transcriptome data. (A)** Overlap of identified circRNAs among the three algorithms using the RNaseR^-^ data. **(B)** Overlap of identified circRNAs between CIRI and find_circ using the RNaseR^-^ data. find_circ-specific candidates can be removed by various filters in CIRI. **(C)** A schematic view of reads mapped to a circRNA region. RNaseR^+^ data were used to validate the circRNA candidates identified based on the RNaseR^-^ data. Dashed rectangle indicates the flanking region adjacent to the junction, which was used to plot the read depth in subgraph E. **(D)** Two typical false positive types identified by the three algorithms. **(E)** Density plot of sequencing depth adjacent to the junction of circRNA candidates. The x-axis represents the relative coordinates of all circRNA candidates with the junction point set to zero; the y-axis represents normalized sequencing depth.

To further evaluate the performance of CIRI, we performed a direct comparison of CIRI with the other two available *de novo* algorithms, segemehl [18] and find_circ [11], using the RNaseR^-^ data set described above. As shown in Figure 4A, CIRI, find_circ and segemehl detected 5,533, 5,542 and 18,418 circRNAs, respectively. Two types of known false positives were compared among the seven subgroups divided according to the overlap of the three predictions, and three subgroups of CIRI (I, III, V) have the lowest FDRs for both types (Figure 4D). Since find_circ provides the junction reads for each predicted circRNA, we used the mapping information of these junction reads, including PEM, to thoroughly analyze the discrepancy between CIRI and find_circ. Amongst the 1,904 candidate circRNAs predicted by find_circ but not by CIRI, 1,783 (about 94%) were indeed filtered by CIRI due to a lack of PCC signals or PEM support (Figure 4B). We also visualized the top five most abundant circRNA candidates solely detected by CIRI or find_circ, and found that at least four of the five CIRI-specific circRNAs have a considerable number of reads mapped after RNase R treatment. In contrast, none of those predicted by find_circ seem to be reliable (Figure S9 in Additional file 1).

To estimate other unknown types of false positives, we utilized the RNaseR^+^ data set as an indicator of prediction reliability. *Bona fide* circRNAs are resistant to RNase R treatment and supposed to have high sequencing depth inside a circular junction (Figure 4C). Therefore, we calculated the normalized sequencing depth adjacent to the junction for each predicted circRNA, which to some extent can reflect the reliability of circRNA prediction. As shown in Figure 4E, circRNAs identified by all three algorithms have a clear pattern of decreased sequencing depth outside the junctions and increased sequencing depth inside the junctions. Similar patterns were also observed in all CIRI identified categories. In contrast, a large proportion of circRNA candidates solely detected by find_circ or segmenhl have no read support inside the junctions (Figure 4E; Table S2 in Additional file 1). Taken together, these results further indicate the advantage of CIRI over the other two algorithms.

### CIRI detects more circRNAs from real data sets from four cell types compared with a previous report

We applied CIRI to paired-end sequencing data generated from CD19+, CD34+, and HEK293 cells and neutrophils. CIRI detected 3,001 circRNAs versus 1,951 detected by Memczak *et al.* [11] from the same data sets (Figure S10 in Additional file 1). Among them, 1,290 overlapping circRNAs identified by both methods comprise about two-thirds of the total number detected by the latter and 43% of CIRI detection results. We focused on *bona fide* circRNAs validated in the Memczak *et al*. study by testing their resistance to RNase R degradation followed by northern blotting. Remarkably, all 22 validated circRNAs (including CDR1as), which vary in abundance, cell type, chromosome origin and spanning distance along the reference, were 100% detected by CIRI.

Subsequently, 661 assumed circRNAs not detected by CIRI were further explored. We selected the top 10 most abundant ones with at least 63 supporting reads, and manually checked the mapping details of all relevant reads. As shown in Figure S10 in Additional file 1, 6 of the 10 assumed circRNAs have adjacent collinear exons that are identical or highly similar to the assumed junction exons. For example, the most abundant one, has_circ_002179, which was described as a circRNA located in chromosome 12 with two junction exons 55 kb away spliced together in chiastic order and supported by 2,842 reads in the Memczak *et al*. study, was shown to indeed be composed of two adjacent exons (1.6 kb away) in canonical order of the *TUBA1B* gene by our further check. Their false prediction may be accounted for by a 61 bp identical sequence from a homologous gene, *TUBA1A*, and the high expression of *TUBA1B* linear transcripts. Among the other four candidates, none were supported by paired-end mapping information, suggesting that they are probably false positives and were ruled out by CIRI filtering.

Amongst the 1,711 circRNAs exclusively predicted by CIRI, we found 526 of them were also detected in our sequencing data from HeLa cells, of which two were experimentally validated. Both of these circRNAs contain a very short exon, 39 bp and 29 bp, respectively, which act as splice donor or acceptor. We noticed that Memczak *et al.*’s algorithm needed both ends of an unmapped read to be entirely aligned to chiastic positions. When junction reads are aligned to extremely short exons, they are usually split into three segments (Figure 1B). Thus, this short-exon flanking type of circRNA would be missed by their algorithm.

### Nearly 100,000 circRNAs were identified from ENCODE transcriptome data from 15 cell lines

Since CIRI is capable of detecting circRNAs located in distinct genomic regions, including intronic or intergenic regions, we analyzed RNA-seq data sets generated in the ENCODE project to further explore the unknown nature of circRNAs in these regions, which also facilitates a comparison between CIRI and the annotation-dependent algorithm reported previously [10].

**Figure 5:**
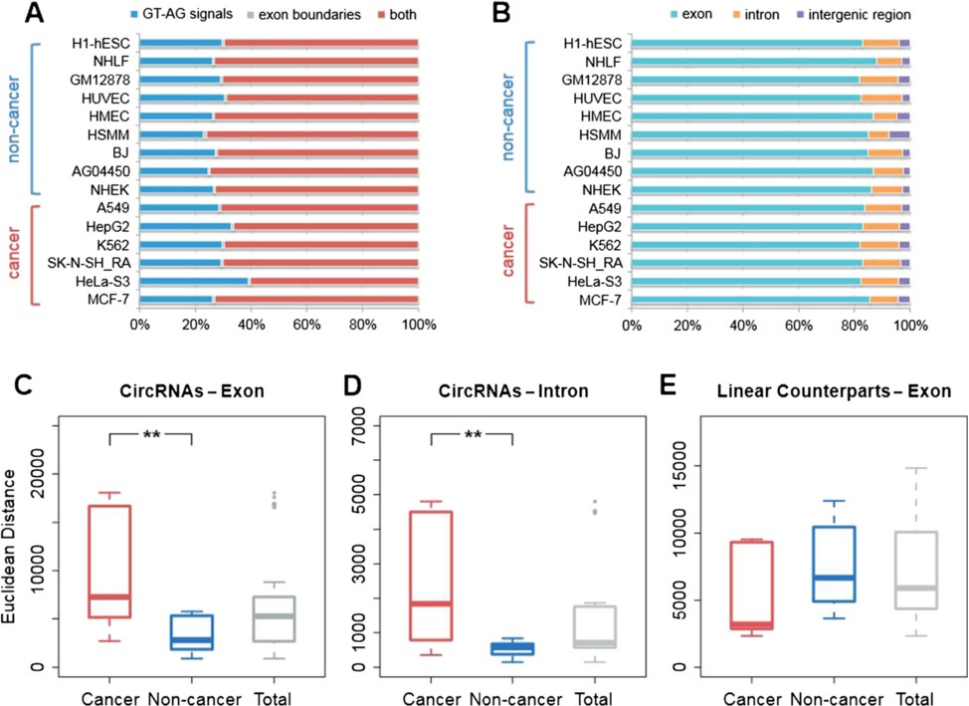
**Cell type-specific expression of circRNAs.** circRNAs identified by CIRI in each cell type were applied as features to calculate distance between a pair of cell types. Euclidean distances between pairs within 6 cancer cell types, 9 non-cancer cell types and all 15 cell types were individually calculated. **(A)** Euclidean distances for 18,894 intronic circRNAs. **(B)** Euclidean distances for 74,719 exonic circRNAs. **(C)** Euclidean distances for corresponding poly-A transcript expression of exonic circRNAs using RPKM values. **(D)** circRNAs identified exclusively by exon boundaries, exclusively by GT-AG splicing signals and by both signatures in each cell type. **(E)** circRNAs located in exonic, intronic and intergenic regions in each cell type.

In total, CIRI identified 98,526 circRNAs from the 15 cell types, of which 18,894 circRNAs (19.2%) are located in intronic regions and 4,913 (5.0%) are located in intergenic regions. Similar distributions were found when making comparisons between cell types. Exonic circRNAs account for the majority of circRNAs detected in each cell type, and circRNAs located in intronic and intergenic regions were also detected in all cell types but in smaller proportions (12 to 19.1%; Figure 5A,B).

We compared the exonic circRNAs detected by CIRI with all circular junctions detected by the annotation-dependent algorithm [10]. Among the 87,195 distinct junctions obtained after removing redundant junctions detected in more than one cell type and ambiguously annotated junctions such as ‘abparts’ from all junctions reported by Salzman *et al.* [10], 27,350 circRNAs were detected by CIRI, which account for about 31% of the 87,195 junctions. It should be noted that 46,866 junctions were indeed indicated after controlling for FDR in their study, which may explain the low percentage of overlap when making a comparison using all junctions. Moreover, a comparison between CIRI and the annotation-dependent algorithm using the subset of validated circRNAs in HeLa cells may provide additional, reasonable explanation for the remarkable difference. Among the 24 validated circRNAs, 21 were also detected by CIRI in the ENCODE data sets, while only 14 were detected by Salzman *et al.* (Table S1 in Additional file 1)*.* Besides all five non-exonic circRNAs that cannot be detected by the annotation-dependent algorithm, three exonic circRNAs were not included in their list - for example, chr1: 117,944,808|117,963,271 is an abundant circRNA detected by CIRI in 12 of 15 cell lines, including HeLa-S3 (Figure S4J in Additional file 1).

To further verify our detected circRNAs in the ENCODE data sets, we performed another comparison between CIRI and a recently published pipeline [16]. In their study, Guo *et al.* [16] set a high cutoff for relative expression of circRNAs (≥10%) and applied their pipeline to 39 ENCODE data sets to derive a conservative catalog. Amongst the 7,058 circRNAs in the catalog, only 8%, 22% and 32% were shared when compared with the three previous studies respectively [16]. However, we found that 75% of them could be confirmed by CIRI in this study (Figure S11A in Additional file 1).

### Cell type-specific expression of circRNAs

To compare the expression of circRNAs across the 15 cell types, we calculated the counts of junction reads for each circRNA normalized by total sequencing reads in each data set as an indicator of their expression levels. Most of the circRNAs had variable expression levels across cell types. As shown in Figure S12 in Additional file 1, significantly distinct expression levels can be observed for the top 50 most abundant circRNAs located in all three types of genomic region. For example, the most abundant one, located in chromosome 20 and annotated as CYP24A1 exonic circRNA, is expressed in only five cell types and is hundreds of times more abundant in cancer cell A549 compared with the other four cell types.

Since the 15 ENCODE RNA-seq samples represent six cancer types and nine non-cancer types, we were able to compare circRNA expression variation between cancer and non-cancer cells. The Euclidean distance was calculated between all pairs of 15 cell types applying exonic and intronic circRNA expression levels as features individually. Comparison of the collection of distances between the six cancer cell types with that between the nine non-cancer cell types showed that the non-cancer distances were significantly smaller than the cancer distances for exonic and intronic circRNA expression (*P* < 0.001 for both, Mann-Whitney U test; Figure 5C,D). We then calculated the corresponding mRNA transcript expression levels of exonic circRNAs using RPKM values and computed distances by the same method. The distances for linear mRNA transcripts show a reverse tendency, that is, the cancer distances are significantly smaller than the non-cancer distances (*P* < 0.01, Mann-Whitney U test; Figure 5E). This indicates that the expression patterns of linear transcripts of circRNA-encoding exons are more similar in cancer cells compared with non-cancer cell types, but the cancer cells appear to have more diverse exonic and intronic circRNA expression profiles.

### More universally shared circRNAs tend to have higher expression levels

**Figure 6:**
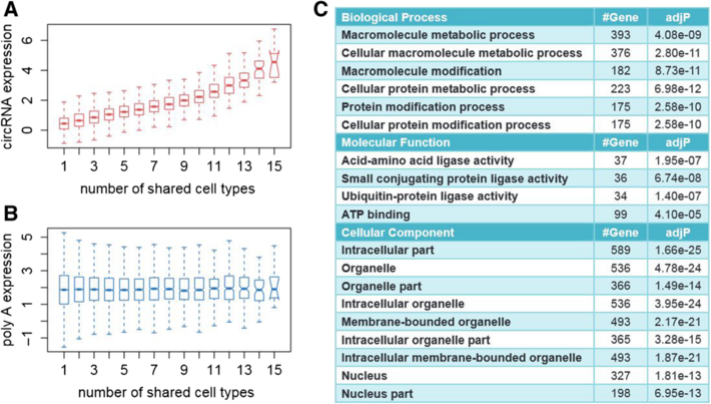
**Comparison of average expression of circRNAs present in different numbers of cell types with their linear counterparts. (A)** circRNAs detected by CIRI are divided into 15 groups according to the number of cell types in which a circRNA is expressed. The expression of a specific circRNA type is represented by the count of junction reads normalized by total sequencing amount in each cell type. The boxplot is generated by R using ln(normalized counts). **(B)** Linear gene expression levels are compared in a similar way as a control. We summarized expression of corresponding genes of exon circRNAs in each cell type using RPKM values recorded in a GTF file. The boxplot was generated by R using ln(RPKM). **(C)** Gene Ontology enrichment analysis for 966 gene IDs of exonic circRNAs expressed in more than 10 cell types.

The annotation information provided by CIRI also facilitates further study on the relationship between the universality of circRNAs and their expression. We first reviewed the output of CIRI for each data set from the 15 cell types to summarize the number of cell types in which an exonic circRNA is detected, and then calculated the average expression level of the circRNAs across the cell types using the counts of junction reads normalized by total sequencing amount of the cell type. Interestingly, we found that circRNAs present in more cell types have significantly higher expression levels than those present in fewer cell types (Figure 6A). To determine whether such elevated expression is caused by the linear mRNA expression background, we then performed a similar analysis on linear gene expression corresponding to each exonic circRNA. The RPKM value for each gene was obtained from a GTF file downloaded from the ENCODE website and the results showed no significant variation in linear gene expression (Figure 6B).

As high abundance and universal expression of genes often suggest important function, we further performed gene set enrichment analysis for the universally expressed circRNAs. We applied Gene Ontology and KEGG (Kyoto Encyclopedia of Genes and Genomes) enrichment analyses to a total of 966 gene IDs of exonic circRNAs present in more than 10 cell types [20,21]. As shown in Figure 6C, significantly enriched Gene Ontology categories are related to a broad range of biological processes, such as metabolic and modification of macromolecule, and molecular functions, such as protein and amino acid ligase activity. KEGG enrichment analysis also points to protein-related processes such as ubiquitin-mediated proteolysis and protein processing in endoplasmic reticulum (Table S3 in Additional file 1).

### Running time and memory use

We determined the running time of CIRI as applied to different data sets. As shown in Figure S13 in Additional file 1, running time increases with file size and number of circRNAs detected. CIRI is fast for small data sets (for example, <5 Gb RNA-seq data) and spends less than half an hour on a 7.5G SAM file using high stringency. It took longer to process large SAM files, but no more than 24 hours was needed even when processing deep sequencing data such as ENCODE data sets (for example, 14.8 hours when processing an 84 Gb SAM file for human skin fibroblast cell line BJ). We simultaneously observed the memory use of CIRI within the running time and found the peak memory cost is about 20% of the SAM file size.

## Discussion

CIRI provides an annotation-independent approach for circRNA detection by employing a *de novo* algorithm. Considering there is little knowledge and few hypotheses on the mechanism of formation of circRNAs at present, this approach can detect novel circRNA candidates for experimental validation and hypothesis generation. In particular, CIRI has the following indispensable advantages over annotation-dependent algorithms: (i) it is able to detect circRNAs transcribed from intronic or intergenic genomic regions; (ii) and it is applicable to sequencing data of eukaryotes that are not well annotated and or even with no annotation.

### Unbiased detection of circRNAs and false discovery rate control in CIRI

Compared with a canonical transcript, circRNA is mainly characterized by its circular junction. Exhaustive identification of junction reads facilitates precise estimation of circRNA abundance based on junction read coverage, and more importantly, detection of certain circRNAs, especially low-abundance ones. Thus, all existing algorithms focus on junction read detection. Salzman *et al.* [10,13] constructed a custom database for all possible exon pairs based on existing annotations to identify reads indicating a non-canonical exon order. Memczak *et al.* [11] and Guo *et al.* [16] adopted an end-to-end alignment to find all incompletely mapped reads combined with an additional two-segment alignment to determine possible junction sites. All of these algorithms were applied to certain data sets in previous studies, which successfully identified plenty of circRNAs. However, based on extensive observations on both simulated and real data sets, we found that different circRNAs may generate various junction read types and a significant proportion of junction reads could be missed by the algorithms mentioned above. For example, short-exon flanking circRNAs generate three-segment junction reads rather than two-segment ones, and non-exonic circRNAs are not detectable using annotation-dependent algorithms. The short segment of an unbalanced junction read may lead to a non-unique alignment, which also challenges the existing algorithms. Considering the complexity of junction reads, CIRI collects and compares raw mapping information for all split alignments of a read to find paired chiastic clipping signals instead of artificially dividing an incompletely mapped read into two parts or aligning all reads to a custom database constructed based on *a priori* assumptions. As the PCC signal is not restricted by read length or mapping segment counts and is also independent of annotation, it should be more reliable for junction detection.

On the other hand, other natural or artificial mechanisms can also produce junction-like reads [15]. Therefore, a filtering system able to rule out false positives from complicated transcriptome sequencing data is necessary. PEM, GT-AG splicing signal/exon boundary and multiple mapping filters individually utilize several sequence and structure features, and ensure CIRI can more specifically predict circRNAs. For example, homologous genes or repetitive sequences in the reference genome can generate erroneous mappings for a split read and thus lead to false positives in other algorithms (Figure 1D; Figures S10 and S11 in Additional file 1). We found that 42 candidates (7% of the total predictions) from the mouse data sets in Guo *et al.* [16] can be attributed to erroneous mappings. In contrast, CIRI could avoid such false predictions owing to its PCC signal and efficient filtering strategies.

We also performed a further analysis on the efficiency of the false positive filtering in CIRI using chimeric RNA as a control. Chimeric RNA could originate from the same strand or different strands of a chromosome and the former may result in false positives because of their similar chiastic mapping to a circRNA. Since the false positive rate resulting from such chimeric RNAs is hard to calculate directly, we utilized chimeric RNA with strand-dissimilarity to estimate the effects of the former on circRNA detection as well as the efficiency of a certain filter for such false positives. We modified CIRI to detect false PCC signals from a fake junction read with its two ends mapping to different strands but kept all of the filters unchanged. As shown in Figure S14 in Additional file 1, two types of chimeric RNAs from different strands significantly decreased in each step. The remaining false positives were about two orders of magnitude fewer compared with the predicted circRNAs from the same dataset, which demonstrated the filtering strategies employed by CIRI are efficient and reliable.

### Intronic or intergenic circRNA fragments provide evidence of non-exonic circRNAs

Previous studies tended to believe that circRNAs are exclusively composed of known exons [10,12,15]. Although Memczak *et al.* [11] and Guo *et al.* [16] predicted non-exonic circRNAs, they did not validate any of them except the well-known CDR1as, which is an intergenic circRNA by our definition, and no details for the candidate non-exonic circRNAs were provided. Most recently, Salzman *et al.* [10] discovered and experimentally validated that a circular isoform of CAMSAP1 contains exon 2, exon 3 and the intervening intron. In this study, we for the first time elaborated on the cases of non-exonic circRNAs as well as ICFs. All the identified ICFs were flanked by GT-AG splicing signals, and they are discontinuously selected and spliced from intronic or intergenic regions, which suggests that the circRNAs containing these ICFs are not intermediates or mis-splicing products of other transcripts. ICFs can form a circle all by themselves (Figure S5A,B in Additional file 1), while in other cases they combine with known canonical exons (Figure 3; Figure S5B,D in Additional file 1). In both scenarios, ICFs could act as a splice donor or acceptor in circRNA formation, which resulted in the inability of annotation-dependent algorithms to detect them.

It has to be mentioned that an intronic circRNA is different from a lariat or a 'circular intronic long noncoding RNA' [22]. It was reported that the loop portion of lariats may escape degradation by RNase R and even generate 'fake' junction reads called branch point reads [12,23]. As shown in Figure S8 in Additional file 1, RNase R treatment completely removed linear transcripts of MED13L and left three ICFs and a putative lariat. However, the 3′ tip of the lariat was digested while all three ICFs remained intact. Although the reverse transcription product of a lariat can also map to the reference genome in a chiastic order, the alignments are not flanked by GT-AG splicing signals. Instead a single dinucleotide, 'GT', which is the splicing signal of exon 2, can be found within the 5′ end of the lariat.

### Detection of non-exonic circRNA in ENCODE data sets

The ENCODE projects demonstrated that transcription is pervasive across the human genome and most bases (up to 93% of the human genome in some tissues) are included in primary transcripts [24]. Combined with studies on other species, it is believed that the present annotations of genomes cannot completely decipher transcription [25]. Several studies have also revealed the intimate association of many known categories of non-coding RNAs with intronic regions [26-28]. Thus, to limit the detection of non-canonical RNA to known exons using annotation obtained from a conventional understanding of canonical splicing is, to some degree, inadequate.

To identify potential non-exonic circRNAs, we used GT-AG splicing signals in CIRI with 15 ENCODE RNA-seq samples. In order to further examine the rationality of splicing signals as a filter for *de novo* detection, we compared outputs using GT-AG signals and known exon boundaries as filters independently in the 15 data sets. The results showed that a small proportion of circRNAs (less than 1%) were exclusively detected using exon boundaries while at least 23% of additional candidates were exclusively found using splicing signals (Figure 5D), which demonstrated that splicing signals could find more circRNA candidates while failing to detect few exonic RNAs.

Scrutiny of the detection outputs showed that intronic and intergenic circRNA candidates account for a relatively stable proportion of all samples. Further study showed no significant correlation between the intronic and intergenic circRNA candidates and sequencing amount of data sets (data not shown), which strongly suggests that the circRNAs are not the result of noise in RNA-seq data. All the results provide evidence that circRNAs are prevalently transcribed from the human genome, and the *de novo* algorithm employed by CIRI is effective for detection of circRNAs, especially for intronic and intergenic circRNAs. Considering the proportion of non-exonic circRNAs in all detected circRNAs from the ENCODE data sets, they could be pervasive and may provide a new essential source material for studying the biogenesis and function of circRNAs.

### Short-exon flanking and small circRNA detection

According to the Gencode version 18 annotation, about one-third of exons in the human genome are shorter than 100 bp (data not shown), which is the most popular read length in current next-generation sequencing. Considering the prevalence of gene sources of circRNAs, short exons could affect the detection and estimation of circRNA abundance because of the complicated mapping patterns of junction reads that contain the exons. In this study, we identified nearly 800 circRNAs with a short exon (≤70 bp) flanking junctions in our HeLa cell sequencing data. Five of them were experimentally validated and all five were also detected by CIRI in at least one of 19 real data sets generated in previous studies (Table S1 in Additional file 1). In contrast, two existing algorithms detected only one of them when applied to the same data.

circRNAs smaller than 40 bp were reported as major components of circRNA categories in archaea [29]. The same study also demonstrated that a circRNA smaller than the insert size or even read length could possibly remain, though at reduced abundance resulting from the lower probability of reverse transcription occurring over more than one complete circle around circRNAs. CIRI employs a specific detection strategy for these potential circRNAs (Figure 1C), and simulated data showed its efficiency with regard to both sensitivity and FDR (Figure S3C in Additional file 1). When analyzing our sequencing data from HeLa cells, however, CIRI detected few small-sized circRNAs (about 0.15% of detected circRNAs). The small proportion of small-sized circRNAs suggests they may be lost in the process of library construction or they are rare species in HeLa cells.

### Optimal choice of parameters in CIRI

Considering CIRI may be applied to various types of RNA-seq data, we provide our recommendations for parameter choice according to the performance of CIRI on simulated data (see Results). Single-end reads result in higher FDRs compared with paired-end reads because of the lack of PEM information as one of the filters when using default parameters. Thus, we provide two parameters (-u, -b) to set mapping quality thresholds, which is able to control FDR within similar levels with paired-end detection but with reduced sensitivity. Although short read length may lead to low sensitivity, alteration of parameters (-k and -T) for BWA-MEM to allow alignments for low mapping scores can improve performance for this type of sequencing data. We also provide stringency parameters based on read counts of a circRNA candidate. A circRNA candidate will be output only when two distinct types of PCC signals support its junction using high stringency, which may leave some less abundant circRNAs undetected due to frequent read duplication in RNA-seq data, while low stringency requires CIRI to output every circRNA candidate regardless of supporting read counts or PCC signal types.

## Conclusion

In this study we propose a novel algorithm, CIRI, that is able to detect circRNAs in a genome-wide range, including intronic and intergenic circRNAs. It does not require RNA-seq data generated after a circRNA enrichment step, such as RNase treatment, or an annotation file as input, and it is applicable to almost all commonly generated read lengths in various sequencing platforms. Systematic filtering in the algorithm ensures a low false positive rate without sacrificing the sensitivity of detecting non-exonic circRNAs and small circRNAs. Extensive simulation studies showed that CIRI has an excellent and unbiased performance with regard to both sensitivity and FDR. A detailed analysis of CIRI output from ENCODE RNA-seq data also revealed new characteristics of circRNAs, including the prevalence of intronic and intergenic circRNAs, which suggests our approach will be a powerful tool for detection and annotation of circRNAs and helpful for further exploration of circRNAs. Since the knockout of ICFs in non-exonic circRNAs does not affect their linear counterparts, the intronic and intergenic circRNAs detected in this study provide good targets for further functional studies.

## Materials and methods

### Algorithm

#### Detection of balanced junction reads based on paired chiastic clipping signals

CIRI requires two types of files, a FASTA formatted reference sequence and a SAM alignment generated by the BWA-MEM algorithm [17], which implements a local alignment and outputs primary alignments for all segments of a query read that separately map to the reference.

CIRI analyzes all alignment records of each read in the SAM file. Briefly, two segments of one read that indicate a circRNA junction should be aligned to the reference genome in a chiastic order (Figure 1A). CIGAR values reflect the junction features in the form of upstream xS/HyM and downstream xMyS/H, where x and y represent the number of mapping (M), soft clipping (S) or hard clipping (H) bases. A typical junction has pairs of such perfectly corresponding alignment records, which we named 'paired chiastic clipping signals', though sequencing errors or fortuitously matching bases may obscure the junction boundary between two separately aligned segments. Notably, some junction reads have a much shorter segment flanking the junction compared with the other segment, which we term an unbalanced junction read. Because the short segment (<19 bp using the default parameter of BWA-MEM) is ignored by the aligner to prevent multiple mapping or erroneous mapping, such junction reads lack one of the necessary clipping signals in the SAM alignment. Therefore, we focus on detection of balanced junction reads in this step and detect unbalanced ones in the following step.

Besides typical junction reads with two segments, more complicated features of several special circRNAs can also be identified. First, if the exon flanking the junction of a circRNA is shorter than the read length, some junction reads of the circRNA may inconsecutively map to the reference in a three-segment style, where two segments map to two exons flanking the junction and the third segment maps to the proximal part of the exon adjacent to the short flanking exon contained in the circRNA (Figure 1B). Second, circRNAs smaller than the read length may also align to the reference in another form of the three-segment style, where two terminal segments separately overlap the terminal parts of the area where the middle segment aligns (Figure 1C). In both situations, CIGAR values show PCC signals in the style of xS/HyMzS/H and corresponding (x + y)S/HzM and/or xM(y + z)S/H, and CIRI checks mapping positions to differentiate the two situations.

If CIRI detects CIGAR values for alignments from the same read that correspond to each other as described above, it then checks the strand information and mapping positions in the SAM alignment. If two segments align to the same chromosome and strand and the distance between them along the genome reference is reasonable, the read is considered as a candidate junction read with positive PCC signal. Strand information and mapping positions are also used to determine the tentative boundaries of segments in the candidate junction read in this step.

#### Filtering of junction reads based on paired-end mapping and GT-AG splicing signals

CIRI utilizes PEM information if available for preliminary filtering of false positive PCC signals. Because two segments of a *bona fide* junction read in theory represent termini of the range where all circRNA reads align, a candidate junction read is considered to indicate a circRNA only when its paired read aligns within the region of the putative circRNA range on the reference genome that segments of the junction read indicate (Figure 1D). For single-ended reads, this step is omitted.

GT-AG signals are major splicing signals in eukaryotic transcription and are used for circRNA detection in CIRI. CIRI loads reference sequences to check whether AG and GT dinucleotides (or reverse complementary dinucleotides CT and AC) flank segments of a junction on a genome (Figure 1D). Due to the ambiguity of junction boundaries identified from alignments, GT and AG signals are accepted if both deviating from the tentative boundaries in the same direction and at the same distance along the reference sequence. Additionally, considering splicing signals for minor introns such as AT-AC and other possible situations where GT-AG splicing signals are not applicable, CIRI can also extract exon boundary positions from a GTF/GFF annotation file provided by users and use them as a complementary or alternative filter for false positives (-x and -a parameters). Candidate junction reads not supported by splicing signals or exon boundaries are filtered out. The tentative junction boundaries are modified and determined according to loci of GT-AG signals or exon boundaries in each junction read. Another optional filter (-E) is also available, here to remove false junction reads based on searching identical sequences on the reference genome. The circRNA balanced junction reads are clustered and recorded subsequently according to their junction loci.

#### Detection of unbalanced junction reads and final filtering based on dynamic programming alignment

After junction loci and flanking segment sequences have been determined, we have adequate information to distinguish an unbalanced junction read as described above from false positives resulting from non-unique mapping of short segments. CIRI scans SAM alignment for the second time and makes a thorough investigation of each read. Information such as CIGAR value, mapping position and mapping quality of a read and its paired read is taken into account. Any read mapping to the related region of a putative circRNA junction in the SAM alignment is aligned with related junction reads identified in the first scan using a dynamic programming algorithm to decide whether the former supports the junction of a circRNA or corresponding linear transcript. Unbalanced junction reads can be detected here even though their flanking segment is not long or informative enough to be uniquely aligned by the aligner.

This step also facilitates a further filter to prevent false predictions resulting from similarity of homologous genes or repetitive sequences (Figure 1D). Briefly, when CIRI detects a read that is highly similar or identical to one of the junction reads but finds its mapping position on the reference is distinct from that of the junction read, CIRI records the read and alignment information. After investigating all reads, CIRI summarizes mapping positions of all related reads of a candidate junction, compares counts and mapping lengths of the reads, and determines whether the reads reliably reflect a circRNA junction and whether the candidate circRNA will be kept in the final output. To set the minimum of relative expression, another optional parameter, --rel_exp, is also available, which calculates the relative expression for each circRNA based on counting junction and non-junction reads around the circular junction.

If a GTF/GFF formatted gene annotation is provided by users, CIRI can refer to the annotation and provide circRNA annotation information in the output file. Three main categories are given according to genome region where a circRNA is located: exon, intron and intergenic region. Notably, a circRNA with one end located in an intergenic or intronic region is categorized as an 'intergenic' or 'intronic' one, no matter where the other end is located. For circRNAs in an exonic or intronic region, we also display related gene information in detail.

### Data sets

#### A simulation tool

CIRI-simulator requires two types of files, a FASTA formatted reference sequence and a GTF or GFF formatted annotation file. First, it loads the annotation file to record gene, transcript and exon information such as position and strand. For each gene, two random numbers are generated for each transcript to determine if the transcript will be selected to generate linear RNA and circRNA reads, respectively. When different transcripts of a gene are selected, exons contained in them will be simulated independently, which mimics alternative splicing in eukaryotes. CIRI-simulator then scans the reference sequence to obtain complete RNA sequences for transcripts selected previously. Simulated reads of linear RNAs and circRNAs are generated randomly by referring to the RNA sequences. Particularly for circRNAs, junction reads are generated simultaneously with reads of other regions by referring to the sequence in the circle. Notably, parameters such as read length, coverage (for both circRNAs and linear RNAs), sequencing error rate, and insert size, can be customized by users. A list of simulated circRNAs will be generated as a FASTQ formatted file to facilitate performance evaluation.

#### Simulated data

We used CIRI-simulator to generate different sequencing data. In detail, human chromosome 1 (hg19) and its GTF annotation file (Gencode version 18, downloaded from [30]) were used as reference and annotation, respectively. We selected read lengths of 40 bp, 60 bp, 80 bp and 100 bp, depth of coverage of 3-, 5-, 10-, 20- and 50-fold, and insert lengths of 200 bp and 350 bp to simulate sequencing reads. Read amounts are determined by sequencing coverage and read length in each data set. For example, about 8 million and 6.5 million reads were generated in simulation data sets with the same coverage settings of circRNAs (10-fold) and linear transcripts (randomly ranging from 1- to 100-fold) for read lengths of 80 bp and 100 bp, respectively. When processing the simulated reads, parameters such as BWA-MEM alignment score minimum of 10, 13, 16, 20, 23, 26 and 30, and max spanning distance of 100 kb, 300 kb, 500 kb, 1 Mb and 2 Mb were also used to estimate the performance of CIRI. Considering alignment errors may occur during BWA alignment for multiple chromosomes, we applied the hg19 whole genome as reference of alignment though only using chromosome 1 as the reference to generate simulated sequencing data. Outputs from CIRI were compared with the lists generated by CIRI-simulator to obtain sensitivity and false positive rate (FDR) using custom scripts.

In the following simulation tests, three poly-A selected sequencing data sets (SRR307005, SRR307006, SRR307007 and SRR307008, 54 bp; SRR317064 and SRR317065, 76 bp; SRR836183, 101 bp) were chosen as blank background. CIRI-simulator was used to generate reads of circular RNAs with corresponding read length at a coverage of 10-fold, using all 24 chromosomes of hg19 and Gencode version 18 GTF annotation file as references and annotation, respectively. Read mapping was performed using BWA-MEM with default parameters except '-T 19', which filters out alignments with score <19 from the output. Parameters of CIRI were as follows: no annotation file provided, using PE mode/SE mode (-p/-s) and default. The mapping quality controller was set to be -u 3 -b 13 only for one test. The parameters of segemehl were as follows: split read mapping (-S), which is necessary for circRNA read mapping and default. Outputs from CIRI and segemehl were compared with the lists generated by CIRI-simulator to obtain sensitivity and false positive rate (FDR) using the same custom scripts mentioned above except for necessary modifications for segemehl in corresponding tests to filter out candidates with a letter 'F', which was described as 'could be wrong' in the segemehl manual, or only keep candidates with supporting counts ≥2 or 3, the former of which was recommended in the additional file of their report [18].

#### Real data

Ribominus RNA-seq data for four cell types (SRP009373, CD19+, CD34+, neutrophils; SRR650317, HEK293) generated in two circRNA-related studies [11,13] were downloaded from the NCBI SRA database. Data sets from both studies comprise paired-end sequencing data, though read lengths are different. Read mapping was performed using BWA-MEM with default parameters except '-T 19'. The SAM alignment records for the two data sets were subsequently analyzed by CIRI separately using PE mode (-p), low stringency (-low), max spanning distance 500 kb (-m 500,000) and Gencode version 18 GTF annotation (-a). We incorporated the outputs to compare them with the prediction results of Memczak *et al.* [11] using a custom script. We also applied CIRI to 15 RNA-seq data sets generated by the ENCODE project and used for circRNA analysis in a more recent report [7]. BWA-MEM and CIRI parameters were the same as described above except for mapping quality thresholds for each segment (-u 3) and total of segments (-b 13) of a candidate junction read. Eleven (SRR060824, SRR192530, SRR192531, SRR765631-SRR765637 and ch12: B-cell lymphoma) out of 18 data sets in the study of Guo *et al.* [16] were used for identification of mouse circRNAs. CIRI parameters were default settings except for SE mode for single-end data. GTF annotation and genome sequences were downloaded from [31] using the latest versions. We incorporated the outputs to compare them with the prediction results of Guo *et al.* [16] using a custom script. Mapping details of candidate circRNAs predicted by the above algorithms were checked using the visual mapping tools inGAP and inGAP-sv [32,33].

#### Direct comparison of CIRI, find_circ and segemehl

We applied CIRI, find_circ and segemehl on our HeLa cell RNA-seq data set without RNase R treatment. Similar parameter adjustments were implemented to rule out effects of different default settings in the three tools. For example, maximum spanning distance was set to 100 kb. Parameters for find_circ were default. For CIRI they were -E, -u 3, -b 13, no annotation provided. For segemehl they were -S, read count ≥2. The putative circRNAs identified by the three tools were further validated by using the mapping depth of corresponding RNase R-treated transcriptomic data. Mapping depth of RNase R^+^ data was calculated for 50 bp upstream to 50 bp downstream of all predicted junctions from the SAM alignment using a custom script independent of the three algorithms. The read depths were then normalized by average read depth inside a junction.

### Experimental validation

#### RNA isolation, ribosomal RNA depletion, RNase R treatment and DNA isolation

Total RNA was isolated using TRIZOL for HeLa cells grown in standard media and conditions. RNA concentration and quality were determined by NanoDrop, Qubit and Agilent 2100. Total RNA was then divided into six replicates and three replicates were depleted of ribosomal RNA using a Ribominus kit (Invitrogen, Carlsbad, CA) according to the manufacturer's instructions. rRNA-depleted RNA from two replicates was incubated at 37°C for 1 hour in 16 μl reaction with 10U/μg RNase R (Epicentre, Madison, WI). Total DNA was isolated from HeLa cells using a DNeasy Blood and Tissue kit (Qiagen, Hilden, Germany).

#### Library preparation and sequencing

Ribosomal RNA and ribosomal RNA-/RNase R-treated samples were used as templates for cDNA libraries. Both libraries were prepared per TruSeq protocol (Illumina, San Diego, CA) and then sequenced on the Illumina HiSeq 2000 platform of the Research Facility Center at Beijing Institutes of Life Science, CAS, with 2 × 101 bp paired reads. The sequencing data were submitted to NCBI SRA with accession numbers SRR1636985 and SRR1636986, and SRR1637089 and SRR1637090 for the two treatments. Another total RNA sample was used for poly-A selected library preparation according to the TruSeq v2 guide.

#### RT-PCR

Poly-A selected, ribosomal RNA- and ribosomal RNA-/RNase R-treated samples mentioned above were used as templates of RT-PCR. cDNA was synthesized using a SuperScript III first-strand kit (Invitrogen) with random hexamers as primers for all three samples. Outward-facing primer sets (Table S4 in Additional file 1) were designed for circular RNA candidates identified by CIRI and PCR reactions were performed for the three cDNA samples and genomic DNA using 35 cycles. PCR products were directly sequenced or sequenced after cloning (for products with insufficient concentration for direct sequencing) to validate circularity.

### Implementation

CIRI is implemented in Perl and has been extensively tested on Linux and Mac OS X. No other tool is required for using CIRI. CIRI is packaged with CIRI-simulator and is available at [34].

## Additional file

Additional file 1: Table S1. Candidate circRNAs for experimental validation. **Table S2.** Direct comparison of three algorithms. **Table S3.** Top 10 KEGG enrichment analysis for 966 gene IDs of exonic circRNAs expressed in more than 10 cell types. **Table S4.** Outward-facing primers of all validated circRNAs. **Table S5.** Identified circRNAs in ENCODE data sets. **Figure S1.** Performance of CIRI on simulated data sets. **Figure S2.** Performance of CIRI and segemehl on data sets with different read lengths and sequencing coverages simulated by CIRI-simulator. **Figure S3.** Performance of CIRI and segemehl on simulated data sets with real poly-A^+^ data sets as blank background. **Figure S4.** Experimental validation of exonic circRNAs. **Figure S5.** Experimental validation of intronic and intergenic circRNAs. **Figure S6.** PCR validation of one exonic circRNA (chr2:58,311,224|58,316,858, A) and one intronic circRNA (chr10: 60,347,975|60,380,661, B) using one pair of outward primers and two pairs of inward primers (within the circle and out of the circle as control) for each of them in RNase^-/+^, genomic DNA and poly-A samples. **Figure S7.** Reads mapped to the junction of intronic circRNA (chr10: 60,347,975|60,380,661). **Figure S8.** CIRI can distinguish intronic circRNAs from intronic lariats. **Figure S9.** Top five most abundant circRNA candidates solely identified by CIRI or find_circ. **Figure S10.** Comparison of circRNA identification from four cell types between CIRI and the algorithm used in Memczak *et al*. **Figure S11.** Comparison of circRNA identification from human and mouse between CIRI and the algorithm used in Guo *et al*. **Figure S12.** Expression levels of top 50 abundant circRNAs identified by CIRI in 15 cell types. **Figure S13.** Running time of CIRI on different data sets. **Figure S14.** Performance evaluation of CIRI’s filters on both circRNAs and chimeric RNAs.

## Abbreviations

bp: base pair
circRNA: circular RNA
DP: dynamic programming
FDR: false discovery rate
ICF: intronic/intergenic circRNA fragment
KEGG: Kyoto Encyclopedia of Genes and Genomes
PCC: paired chiastic clipping
PCR: polymerase chain reaction
PEM: paired-end mapping
SAM: Sequence Alignment/Map
