# Supplementary material for: CIRI: an efficient and unbiased algorithm for de novo circular RNA identification
Source: Genome Biol. 2015 Jan 13;16(1):4. doi: 10.1186/s13059-014-0571-3 (PMC4316645; doi:10.1186/s13059-014-0571-3)
Supplement: Additional file 1: Table S1. — Candidate circRNAs for experimental validation. Table S2. Direct comparison of three algorithms. Table S3. Top 10 KEGG enrichment analysis for 966 gene IDs of exonic circRNAs expressed in more than 10 cell types. Table S4. Outward-facing primers of all validated circRNAs. Table S5. Identified circRNAs in ENCODE data sets. Figure S1. Performance of CIRI on simulated data sets. Figure S2. Performance of CIRI and segemehl on data sets with different read lengths and sequencing coverages simulated by CIRI-simulator. Figure S3. Performance of CIRI and segemehl on simulated data sets with real poly-A+ data sets as blank background. Figure S4. Experimental validation of exonic circRNAs. Figure S5. Experimental validation of intronic and intergenic circRNAs. Figure S6. PCR validation of one exonic circRNA (chr2:58,311,224|58,316,858, A) and one intronic circRNA (chr10: 60,347,975|60,380,661, B) using one pair of outward primers and two pairs of inward primers (within the circle and out of the circle as control) for each of them in RNase-/+, genomic DNA and poly-A samples. Figure S7. Reads mapped to the junction of intronic circRNA (chr10: 60,347,975|60,380,661). Figure S8. CIRI can distinguish intronic circRNAs from intronic lariats. Figure S9. Top five most abundant circRNA candidates solely identified by CIRI or find_circ. Figure S10. Comparison of circRNA identification from four cell types between CIRI and the algorithm used in Memczak et al. Figure S11. Comparison of circRNA identification from human and mouse between CIRI and the algorithm used in Guo et al. Figure S12. Expression levels of top 50 abundant circRNAs identified by CIRI in 15 cell types. Figure S13. Running time of CIRI on different data sets. Figure S14. Performance evaluation of CIRI’s filters on both circRNAs and chimeric RNAs. [file 13059_2014_571_MOESM1_ESM.pdf]

Supplementary Table 1. Candidates circRNAs for experimental validation.

gene:start_exon|end_exon	chr	start position	end position	region in genome	# junction reads	PCC signal types	detected in 15 cell types[1]		detected in 4 cell types[2]		outward-facing primer PCR validation	
							CIRI	PG	CIRI	NAT	Total RNA	RNase R
PHC3-011:exon5|exon6	chr3	169863211	169867032	exonic	39	25_27_0	y	y	y	y	y	y
HIPK3-003:exon2|exon2	chr11	33307959	33309057	exonic	208	53_53_2	y	y	y	y	y	y
NFATC3-006:exon2|exon3	chr16	68155890	68160513	exonic	61	27_29_1	y	y	y	y	y	y
POLR2A-001:exon9|exon10	chr17	7402358	7402810	exonic	33	19_24_1	y	y	y	y	y	y
FAM120A-004:exon2|exon5	chr9	96233423	96261168	exonic	86	34_37_1	y	y	y	y	y	y
CRIM1-001:exon3|exon4	chr2	36668401	36669878	exonic	66	28_28_2	y	y	n	n	y	y
CRIM1-001:exon2|exon4	chr2	36623757	36669878	exonic	46	17_21_0	y	y	n	n	y	y
PDCD11-001:exon26|exon27	chr10	105197772	105198565	exonic	32	16_20_7	y	y	y	y	y	y
SFMBT2:exon5|exon7	chr10	7318854	7327916	exonic	256	58_58_5	y	y	y	y	y	y
SFMBT2:exon5|exon9	chr10	7285520	7327916	exonic	68	31_30_0	y	y	n	n	y	y
ANKRD36BP2:exon4|exon12	chr2	89082251	89092011	exonic	46	8_17_18	**n**	**n**	**y**	**n**	y	y
RSRC1:exon2|exon3	chr3	157839892	157841780	exonic	125	43_45_3	y	y	y	y	y	y
EXOC6B:exon3|exon6	chr2	72945232	72960247	exonic	92	36_23_15	**y**	**y**	**n**	**n**	y	y
VRK2:exon6|exon10	chr2	58311224	58316858	exonic	56	14_26_14	**y**	**n**	**n**	**n**	y	y
MAN1A2:exon2|exon4	chr1	117944808	117957453	exonic	64	33_33_2	y	n	y	y	y	y
MAN1A2:exon2|exon5	chr1	117944808	117963271	exonic	230	55_53_6	y	n	y	y	y	y
MAN1A2:exon2|exon3	chr1	117944808	117948267	exonic	41	25_23_1	n	y	y	y	y	y
MAN1A2:exon2|exon6	chr1	117944808	117984947	exonic	58	24_24_1	y	y	y	y	y	y
intergenic	chr5	10213603	10224173	intergenic	62	27_25_2	**y**	**n**	**n**	**n**	y	y
intergenic	chr8	128152989	128186609	intergenic	87	37_37_1	**y**	**n**	**n**	**n**	y	y
BICC1:intron	chr10	60347975	60380661	intronic	17	12_8_3	**y**	**n**	**n**	**n**	y	y
MED13L:intron	chr12	116668338	116675510	intronic	67	27_27_1	y	n	y	y	y	y
MED13L:intron	chr12	116674626	116675510	intronic	29	11_17_6	**y**	**n**	**n**	**n**	y	y
BANP:exon8|exon11	chr16	88061089	88071617	exonic	69	31_11_19	y	y	y	n	y	y
CCAT1	chr8	128231055	128231211	exonic	18	13_14_1	y	n	n	n	y	n
DYRK1A:intron	chr21	38792601	38794168	intronic	76	32_34_4	y	n	y	y	n	n/a
POLR2A-001:exon9|exon10	chr17	7402358	7402810	exonic	33	19_24_1	y	n	y	y	n	n/a
PRSS53:intron	chr16	31102096	31102663	intronic	22	15_17_	y	n	n	n	n	n/a
ARID1B:intron	chr6	157357969	157406039	exonic	7	2_5_3	y	y	y	y	n	n/a
ZCCHC11:exon4|exon7	chr1	52959283	52975384	exonic	19	8_13_2	y	n	n	n	n	n/a
GSE1:exon2|exon2	chr16	85667520	85667738	exonic	146	55_51_3	y	n	y	n	n	n/a
DNAH14:exon2|exon10	chr1	225140372	225195246	exonic	31	19_17_1	y	y	n	n	n	n/a
intergenic	chr8	128231055	128239786	intergenic	15	14_9_1	y	n	n	n	y	n
ARHGEF12:exon2|exon3	chr11	120276827	120278532	exonic	18	1_11_10	y	y	n	n	n	n/a

[1] The 15 cell types of ENCODE data sets used for circRNA detection in a previous study (Salzman et al., PLoS Genetics, 2013). See Method for detail.

[2] The 4 cell types used for circRNA detection in previous studies (Memczak et al., Nature, 2013; Salzman et al., PLoS One, 2012): CD19+, CD34+, neutrophils and HEK293.

Supplementary Table 2. Direct comparison of the three algorithms.

Algorithm	CIRI	find_circ[1]	segemehl[2]
Candidate circRNAs detected	5,533	5,542	18,418
Candidates with identical colinear exons	0	64(1.2%)	558(3.0%)
Candidates with two ends from different genes	21(0.4%)	113(2.0%)	92(0.5%)
Total junction read counts	38,644	34,804	89,553
Junction read # per candidate circRNA	6.98	6.28	4.86
Read depth near the left junction	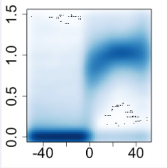	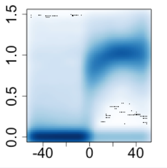	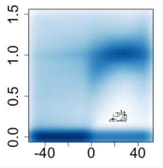
Read depth near the right junction	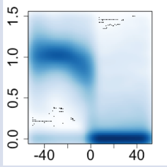	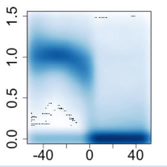	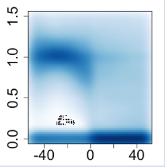

[1] find_circ: algorithm used in Memczak et al., Nature, 2013

[2] segemehl: algorithm reported in Hoffmann et al., Genome Biology, 2014

Supplementary Table 3. Top 10 KEGG enrichment analysis for 966 gene IDs of exonic circRNAs expressed in more than 10 cell types.

Pathway Name	#Gene	adjP
**Ubiquitin mediated proteolysis**	27	1.95e-19
**Protein processing in endoplasmic reticulum**	26	2.85e-16
**Metabolic pathways**	52	1.92e-09
**Pathways in cancer**	25	8.96e-09
**Insulin signaling pathway**	13	1.32e-05
**Colorectal cancer**	9	1.65e-05
**Wnt signaling pathway**	13	2.44e-05
**ErbB signaling pathway**	10	2.76e-05
**Prostate cancer**	10	2.81e-05
**Pancreatic cancer**	9	2.81e-05

Supplementary Table 4. Outward-facing primers of all validated circRNAs.

gene:start_exon|end_exon	chr	start position	end position	outward-facing primer	
				F	R
PHC3-011:exon5|exon6	chr3	169863211	169867032	GAGCTTGGCTTGCCGTTAG	TGTTGTCTCGTCGTCATCGT
HIPK3-003:exon2|exon2	chr11	33307959	33309057	GTTGGATAATCATAGCAGCG	AGGTCCGTGGATAGTTTCTTT
NFATC3-006:exon2|exon3	chr16	68155890	68160513	ATCGAGCCCATTATGAAACTGA	GGACAGAAGCATTGAGCCAC
POLR2A-001:exon9|exon10	chr17	7402358	7402810	ATGCAGACTTTGACGGGGATG	GACCATGGGAGAATGCGGAC
FAM120A-004:exon2|exon5	chr9	96233423	96261168	CGACTAGTAAGCCTATGTCAT	AGTGCATAATCAGAGTCATAC
CRIM1-001:exon3|exon4	chr2	36668401	36669878	CGGACAGCTATGAAACTCAAGTCA	CTGGTTTGCACTCATAGAGGTC
CRIM1-001:exon2|exon4	chr2	36623757	36669878	CGGACAGCTATGAAACTCAAGTCA	CTGGTTTGCACTCATAGAGGTC
PDCD11-001:exon26|exon27	chr10	105197772	105198565	GACGAAAAGCAAAGTAGAAG	AGCGACAAAGTCAATACGTT
SFMBT2:exon5|exon7	chr10	7318854	7327916	CGAACCAGTCAAGTCACGTATG	ATGGCCTCTGAATGGAAATGTAC
SFMBT2:exon5|exon9	chr10	7285520	7327916	CGAACCAGTCAAGTCACGTATG	ATGGCCTCTGAATGGAAATGTAC
ANKRD36BP2:exon4|exon12	chr2	89082251	89092011	TCAGCCAAGATAGACAAGAACT	ATGTCTCCCAGATTGTTGTC
RSRC1:exon2|exon3	chr3	157839892	157841780	GGCATCGATCAAGCAGTAGC	CGGCTGTATGTTCTACTATCTG
EXOC6B:exon3|exon6	chr2	72945232	72960247	AGCAGTGTCGACTACAACAGA	CTGCACTGAAAACTCTGGAA
VRK2:exon6|exon10	chr2	58311224	58316858	TGTCCTGCAATTAGGTATCC	GCCTGAGATCTTCTGTAAATC
MAN1A2:exon2|exon4	chr1	117944808	117957453	TGCTTGGGATAACTATAGGAC	TGTCATTATCTTCTGGGTCTC
MAN1A2:exon2|exon5	chr1	117944808	117963271	TGCTTGGGATAACTATAGGAC	TGTCATTATCTTCTGGGTCTC
MAN1A2:exon2|exon3	chr1	117944808	117948267	TGCTTGGGATAACTATAGGAC	TGTCATTATCTTCTGGGTCTC
MAN1A2:exon2|exon6	chr1	117944808	117984947	TGCTTGGGATAACTATAGGAC	TGTCATTATCTTCTGGGTCTC
intergenic	chr5	10213603	10224173	GTCCTCCACAGTCTTATCTCATT	CCACTCACTTAGGCAAAAAGAA
C10orf90:intron	chr8	128152989	128186609	TAGCTTAGTTCTGCCGTAAAGT	TCACCGCAATGCCAAATGTA
BICC1:intron	chr10	60347975	60380661	GTCCAGTGGGGTTATGAATG	AGCCTCTCATATTCAGAAAAGG
MED13L:intron	chr12	116668338	116675510	TCTGGTTTGACATCACGACG	CCTGACTGAATTGGAAAGGAAT
MED13L:intron	chr12	116674626	116675510	CTTGCAGACAGCGGATGAAACT	GCTCCGGTGTTGCCTGATTT
BANP:exon8|exon11	chr16	88061089	88071617	TACCGAATCAAGCAGAGCATCGA	AGTCGGATTCTGTGATGCCAAATT

Supplementary Table 5. Identified circRNAs in ENCODE data sets.

The tables can be download at

http://159.226.116.227/~fangqing/Site/shared/ENCODE_xls.zip.

Figure S1. Performance of CIRI on simulated data sets. (A) Sensitivity and FDR of varying coverage of circRNAs; (B) Sensitivity and FDR of varying read length of sequencing data (40bp sequencing data is aligned by BWA-MEM with distinct parameters -k 13, -T 13, while other read length with the only parameter: -T 19); (C) Sensitivity and FDR of varying sized circRNAs; (D) Sensitivity and FDR of varying –k parameter of BWA-MEM; (E) Sensitivity and FDR of CIRI using different max spanning distance settings.

Figure S2. Performance of CIRI and segemehl on data sets with different read lengths and sequencing coverages simulated by CIRI-simulator.

Figure S3. Performance of CIRI and segemehl on simulated data sets with real poly-A$^+$ data sets as blank background. (A) Sensitivity and FDR of both tools using default parameter settings; (B) Sensitivity and FDR of segemehl with "F" removed compared with default parameter settings of CIRI; (C) Sensitivity and FDR of segemehl with supporting read counts ≥ 2 and ≥3 compared with default parameter settings of CIRI; (D) Sensitivity and FDR of segemehl with supporting read counts ≥ 3 compared with two parameter settings of SE mode of CIRI.

Figure S4. Experimental validation of exonic circRNAs. Exons used in circRNAs are shown in colored arrows. The linear fragments across the junction were amplified using a pair of outward-facing primers. (G-I) multiple fragments could be amplified because of the presence of alternatively spliced circRNAs. Scissors indicate the splicing sites, which are flanked by GT-AG signals highlighted in red. Sequencing chromatogram of the PCR product across the junction is shown.

Figure S5. Experimental validation of intronic and intergenic circRNAs. ICFs used in circRNAs are shown in hollow arrows. The linear fragments across the junction were amplified using a pair of outward-facing primers. (B, D) multiple fragments could be amplified because of the presence of alternatively spliced circRNAs. Scissors indicate the splicing sites, which are flanked by GT-AG signals highlighted in red. Sequencing chromatogram of the PCR product across the junction is shown.

Figure S6. PCR validation of one exonic circRNA (chr2:58,311,224|58,316,858, A) and one intronic circRNA (chr10: 60,347,975|60,380,661, B) using one pair of

outward primers and two pairs of inward primers (within the circle and out of the circle as control) for each of them in RNase -/+, genomic DNA and poly-A samples.

Figure S7. Reads mapped to the junction of intronic circRNA (chr10: 60,347,975|60,380,661). Reads with the same color are pair-end reads.

Figure S8. CIRI can distinguish intronic circRNAs from intronic lariats. (A) Coverage plot of one exon and three ICFs contained in chr12:116,668,338|116,675,510 in the two samples (red: junction reads identified by CIRI in $RNaseR^-$; blue: junction reads identified by CIRI in $RNaseR^+$; grey: other reads). Scissors indicate the splicing sites, which are flanked by GT-AG signals highlighted in red. (B) Two circRNAs' structures and split mapping of junction reads. (C) After RNase R treatment, 3' tip of the lariat is digested, and the remaining sequence forms a circle. However, when the junction read is mapped back to the reference sequence, no flanking GT-AG signal is present.

Figure S9. Top 5 most abundant circRNA candidates solely identified by CIRI or find_circ. X-axis represents the coordinates on the reference genome; y-axis represents the sequencing depth.

Figure S10. Comparison of circRNA identification from four cell types between CIRI and the algorithm adopted in Memczak, et al. (A) Overlap between the circRNAs identified by both algorithms. (B) The most abundant 10 candidates identified by Memczak, et al but not by CIRI are listed in the table. They can be mainly divided into two groups: One group of candidates seem to be false positives due to multiple mapping of a segment of a read to identical regions, while the other group of candidates are not supported by PEM. (C) An example shows the wrong mapping of one segment to repetitive regions. (D) An example shows the falsely discovered circRNA that is rejected by PEM.

Figure S11. Comparison of circRNA identification from human and mouse between CIRI and the algorithm adopted in Guo, et al.

(A) Overlap between the circRNAs identified in human cell types by both algorithms (Guo, et al: 39 cell types [15]; CIRI: 15 cell types, see Methods). (B) Overlap between the circRNAs identified in mouse data sets by both algorithms (Guo, et al: 18 data sets [15]; CIRI: 11 data sets, see Methods). (C-D) Two examples show the erroneous mapping of one segment to repetitive regions. (E) Among 279 candidates

identified by Guo, et al but not by CIRI, 42 have identical colinear exons and were filtered out by CIRI considering their mapping information.

Figure S12. Expressions of top 50 abundant circRNAs identified by CIRI in 15 cell types. The expression of a specific circRNA type is represented by count of junction reads normalized by total sequencing amount in each cell. The normalized junction read counts is proportional to area of corresponding bubble. The color of bubbles corresponds to category of cell type: red represents cancer cells; blue and green represent non-cancer cells; red and blue colors ranging from darkness to lightness represent endodermal, mesodermal and ectodermal cells, respectively; green color represents the only pluripotent cell type H1-hESC; black color represents average expression in cell types where a circRNA expresses. Y-axis: top 50 most abundant circRNAs locating in exonic, intronic and intergenic regions are marked in black, blue and red, respectively.

Figure S13. Running time of CIRI on different data sets.

Figure S14. Performance evaluation of CIRI's filters on both circRNAs and chimeric RNAs. (A) Chimeric RNA sequences which are split-aligned to different strands can be classified into two categories according to their mapping signatures on the reference genome; (B) The efficiency of CIRI's filters on removing false positives when detecting circRNAs and chimeric RNAs from the HeLa cell transcriptome data. Y-axis represents the number of remaining candidates in each filtering step.

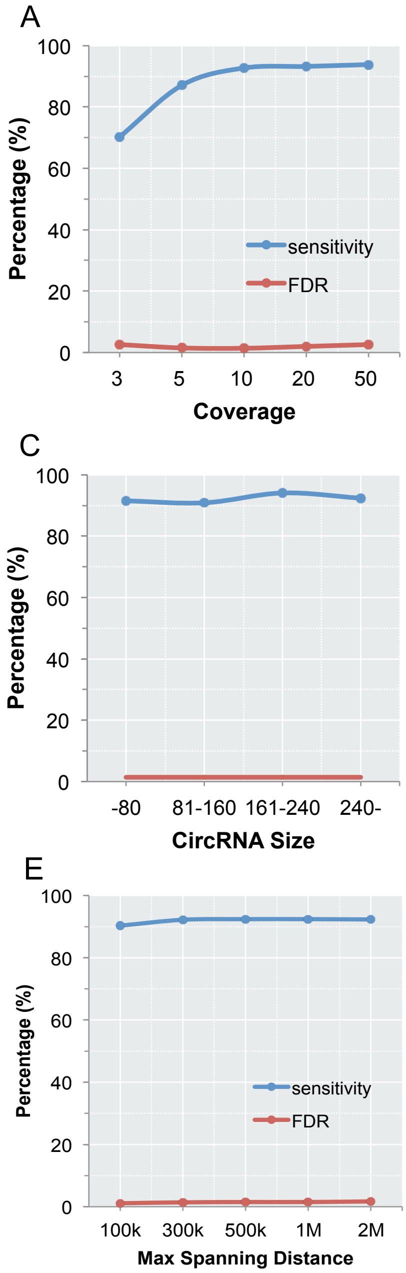

Suppl. Figure 1

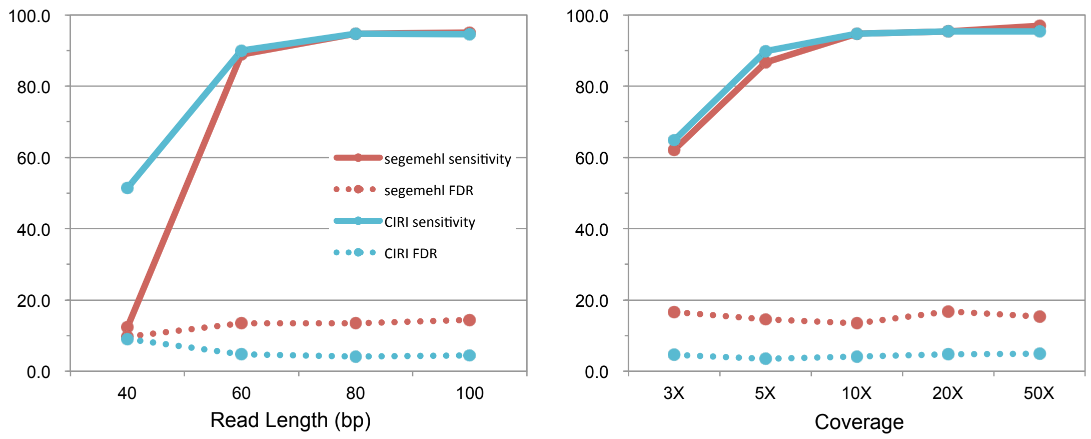

Suppl. Figure 2

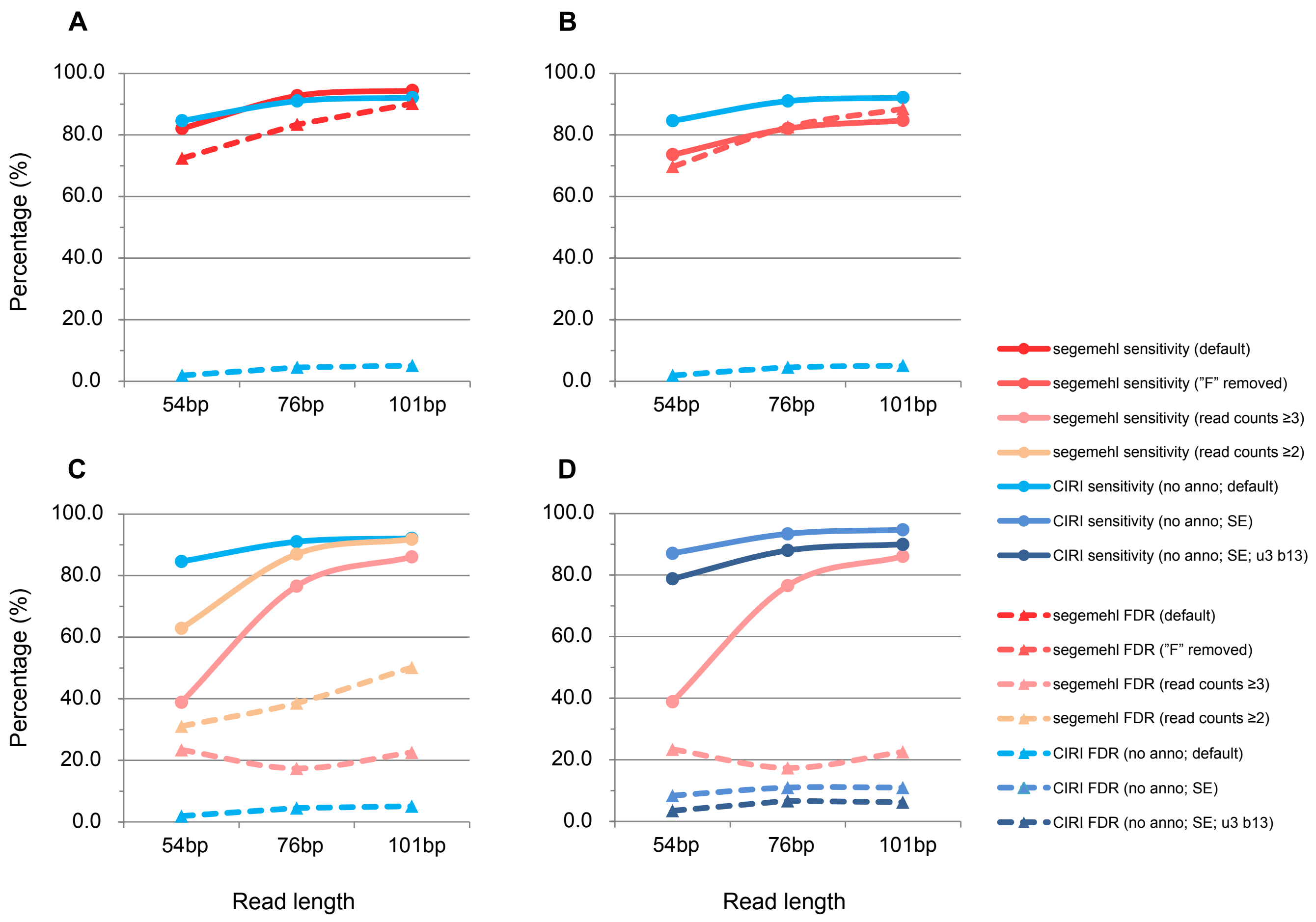

Suppl. Figure 3

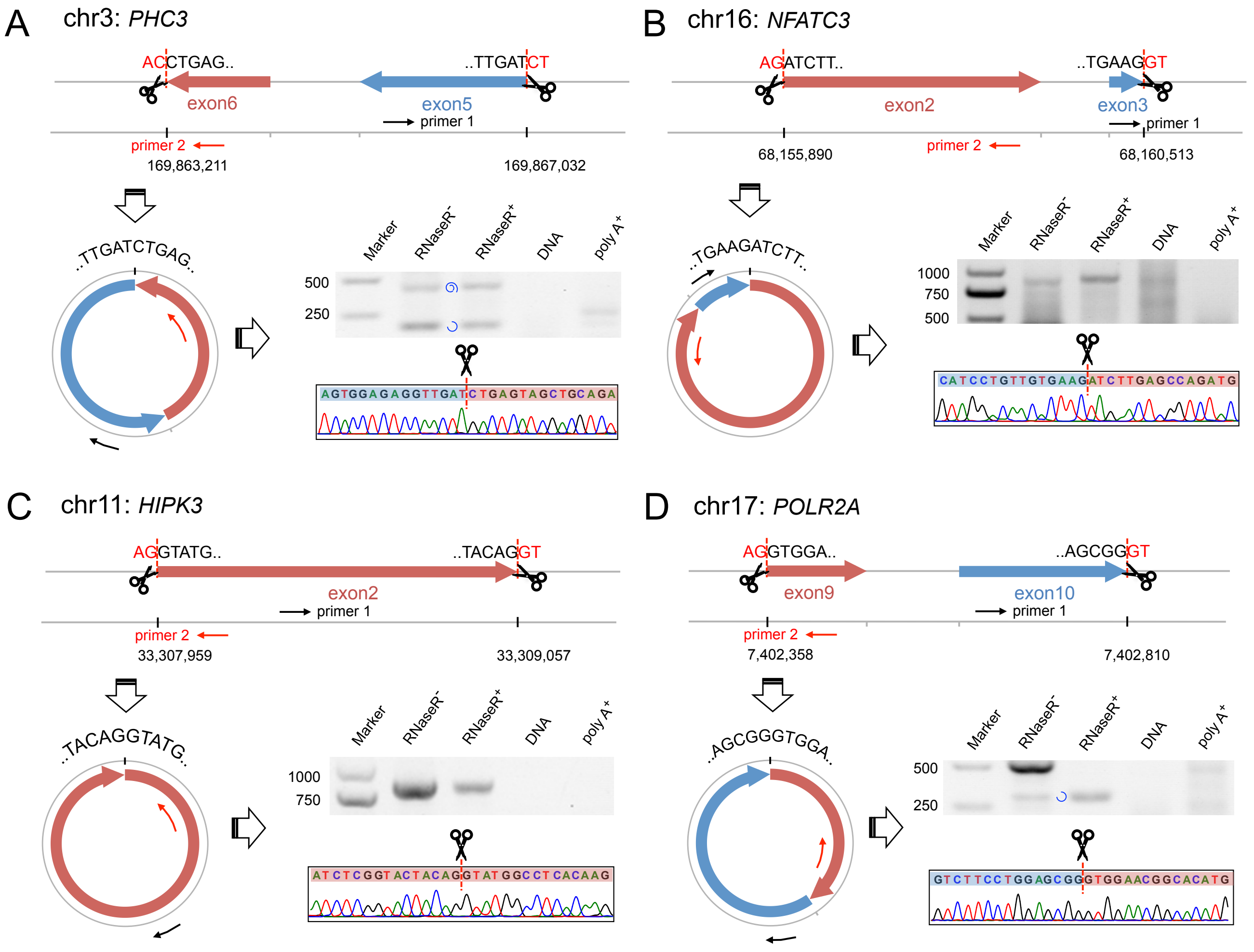

Suppl. Figure 4

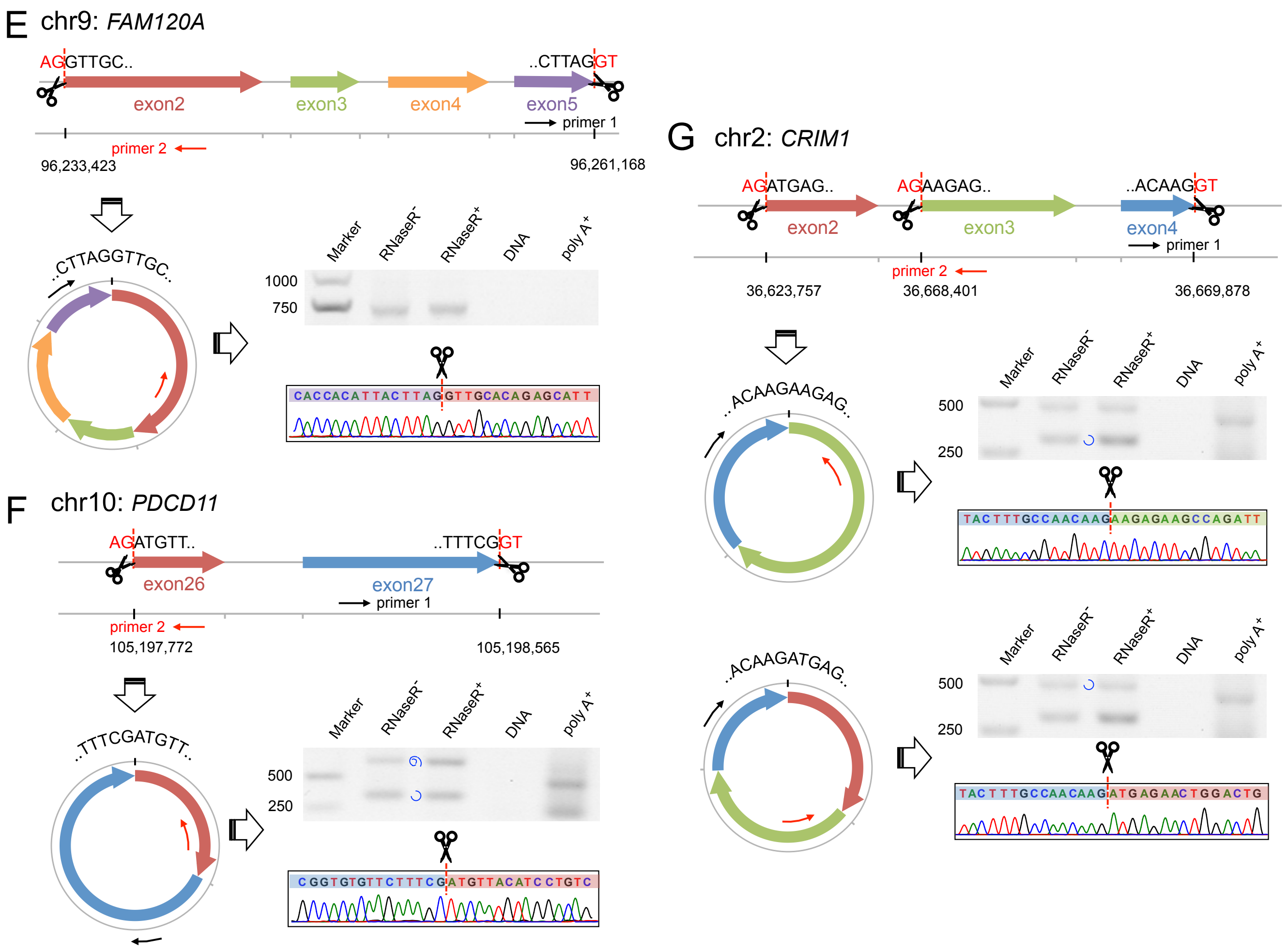

Suppl. Figure 4 – *continued*

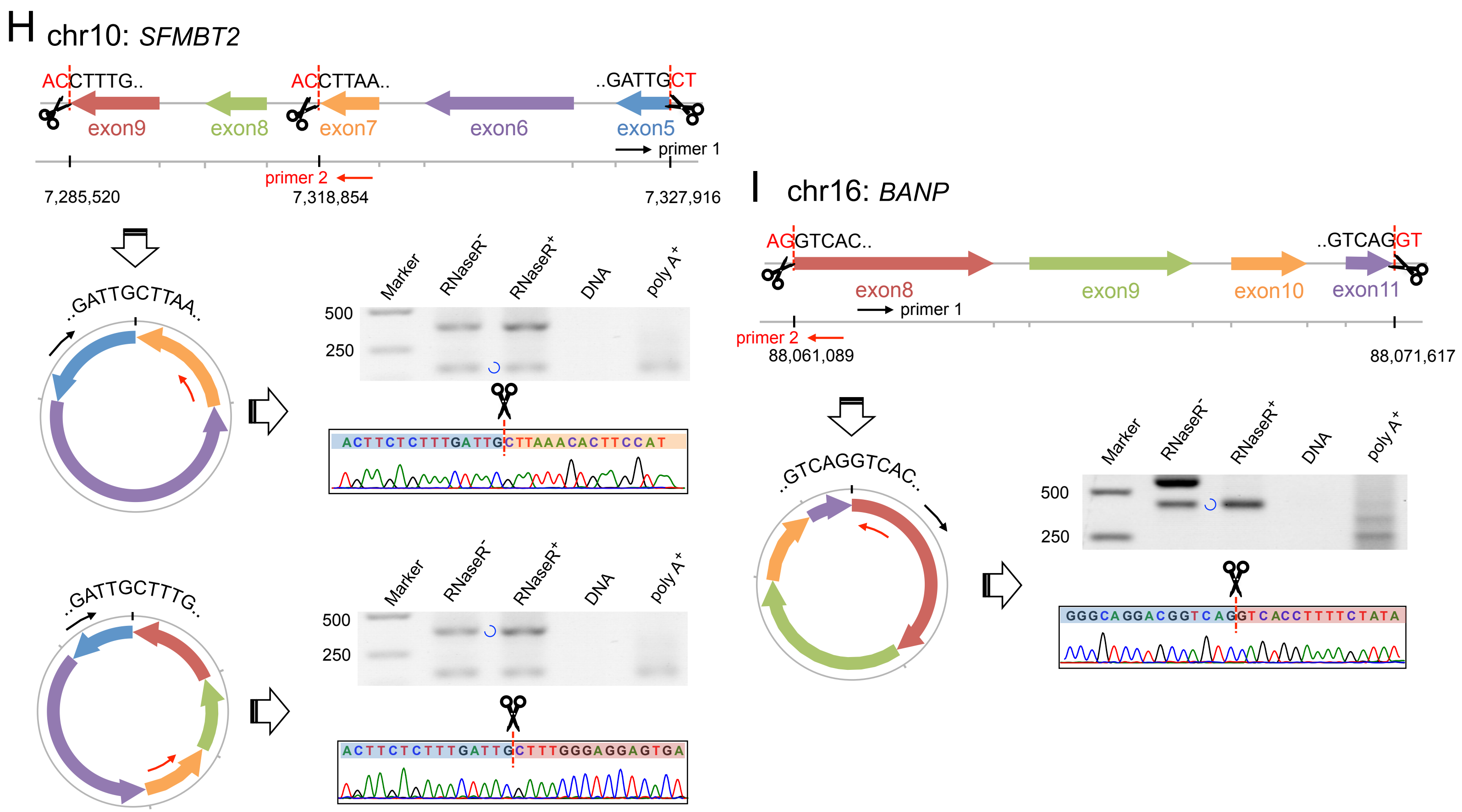

Suppl. Figure 4 – *continued*

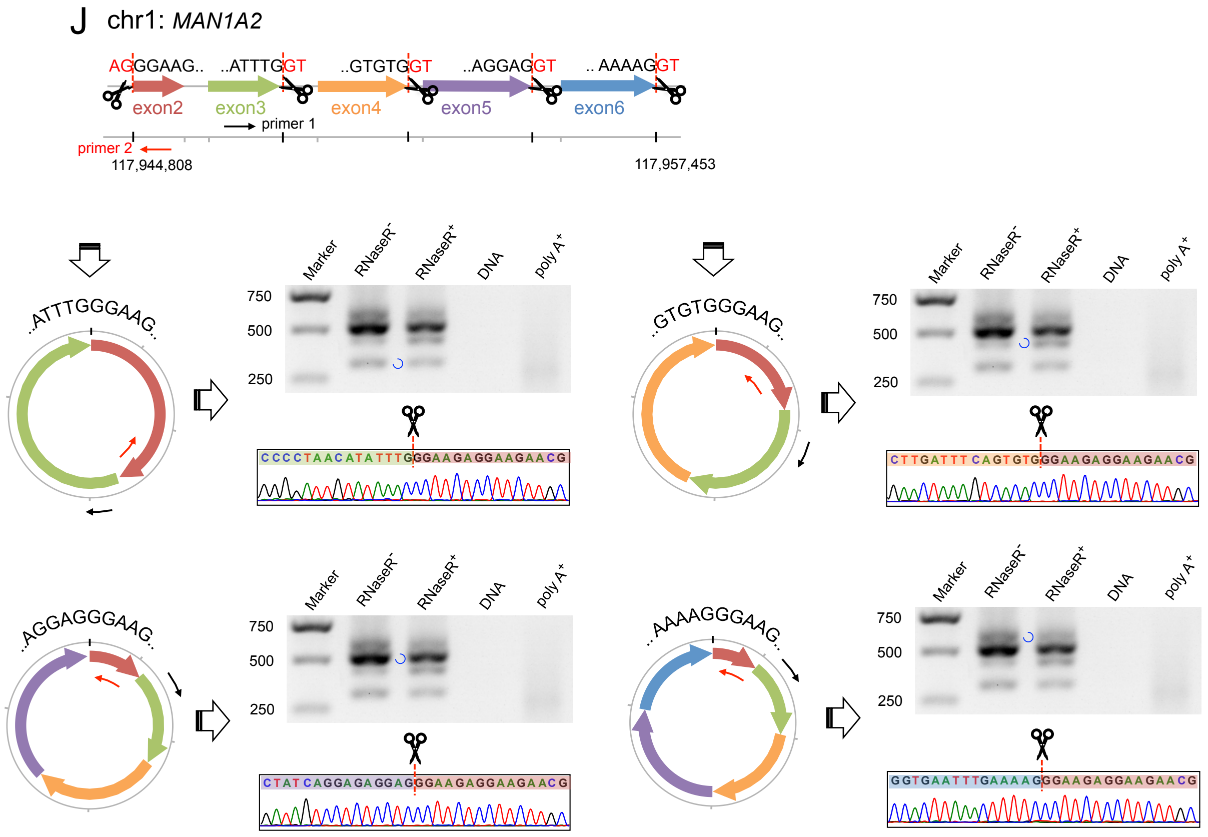

Suppl. Figure 4 – *continued*

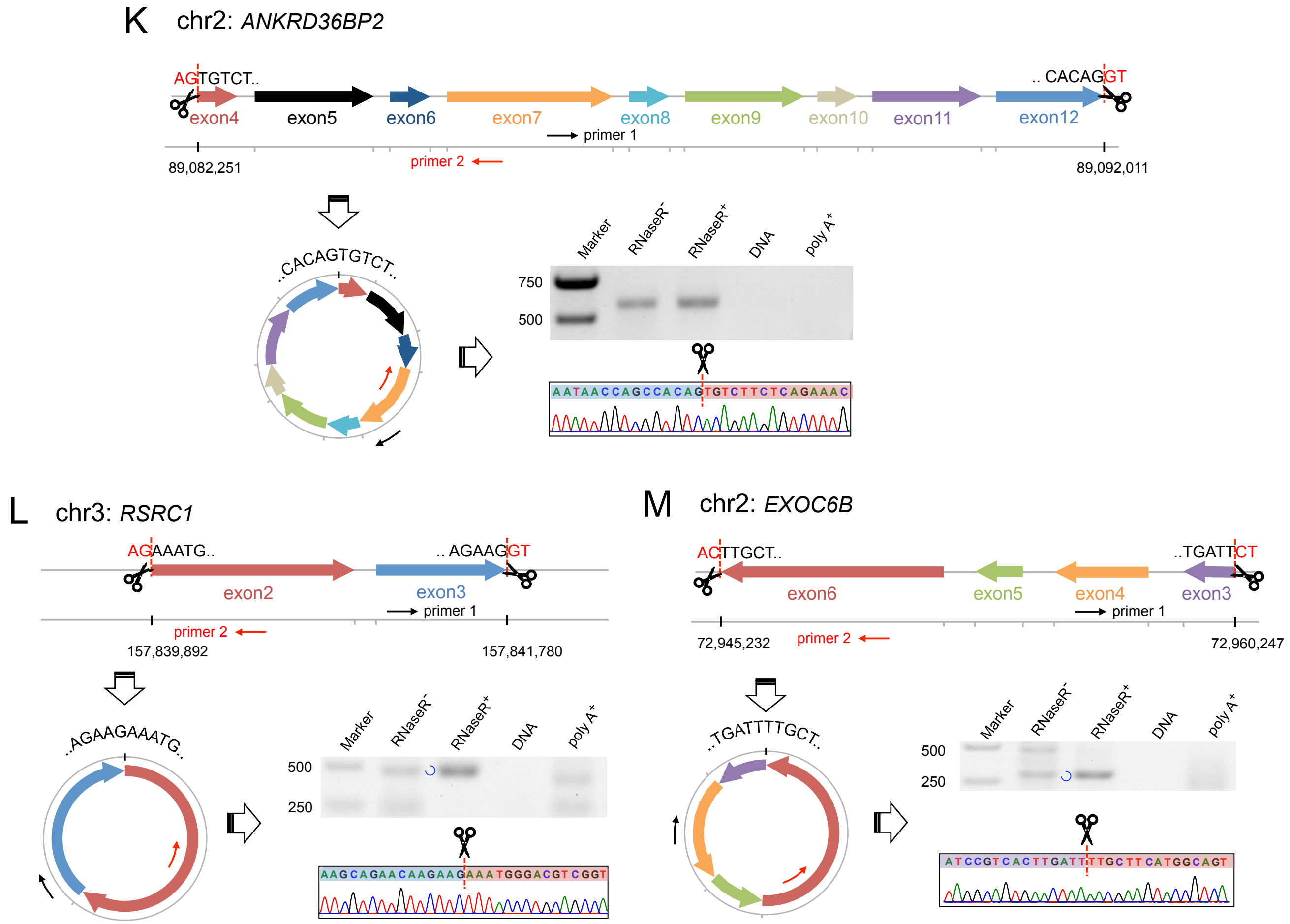

Suppl. Figure 4 – *continued*

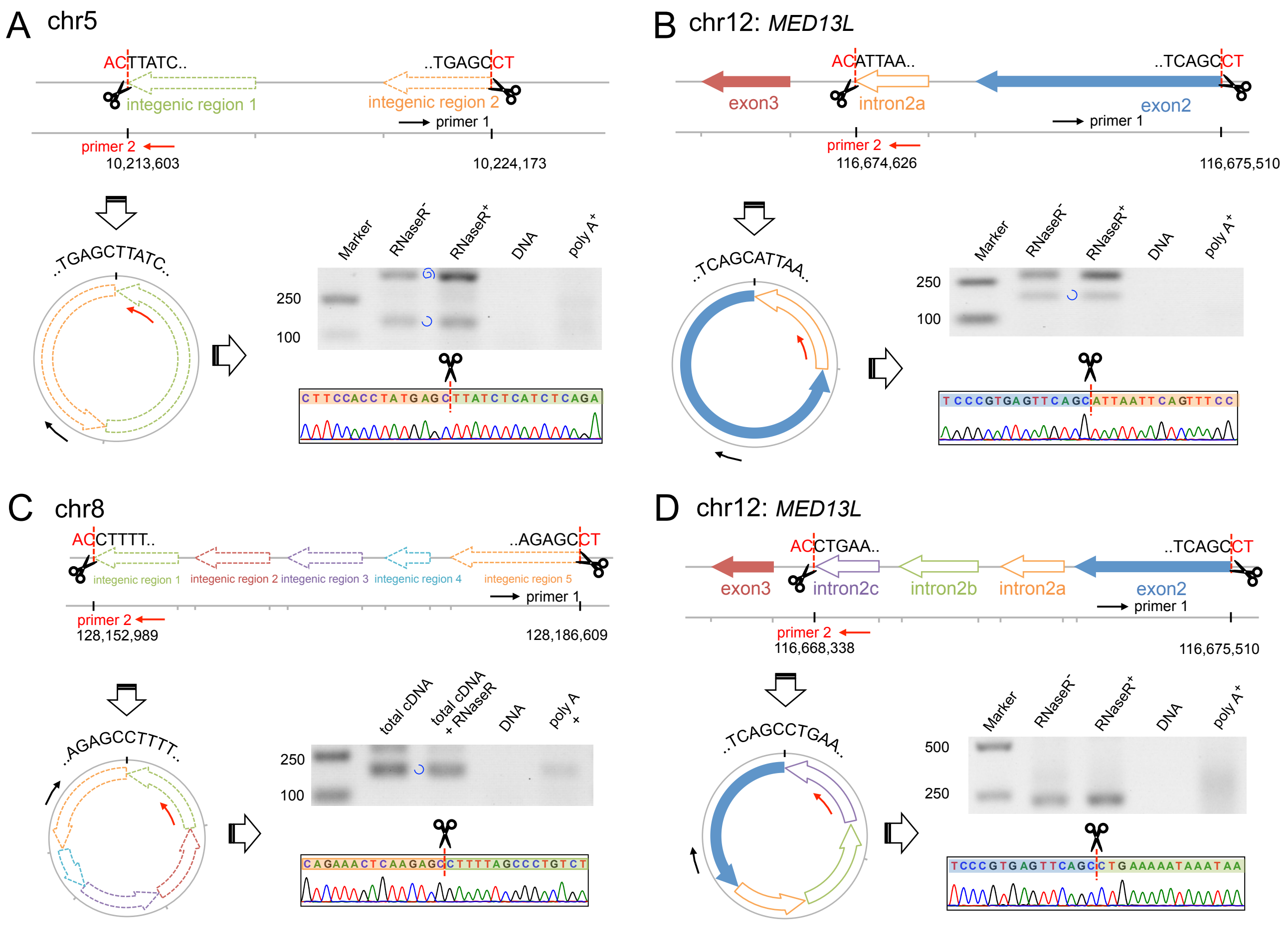

Suppl. Figure 5

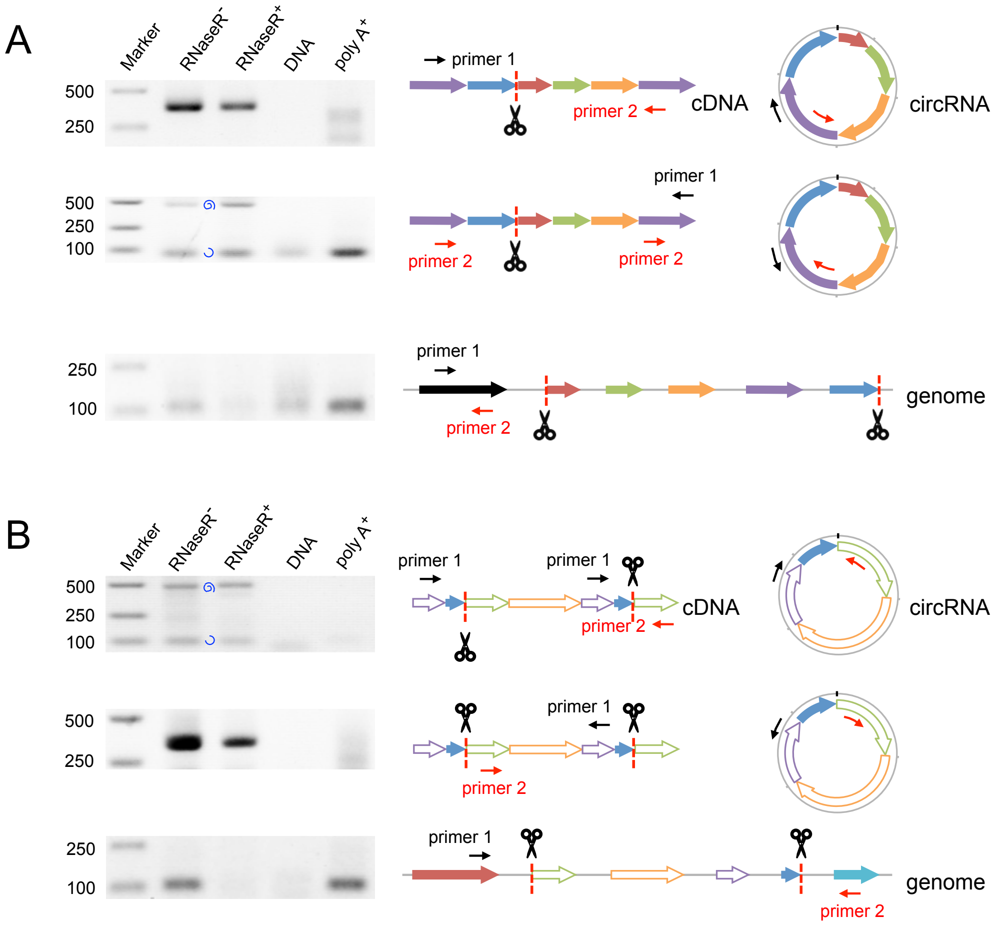

Suppl. Figure 6

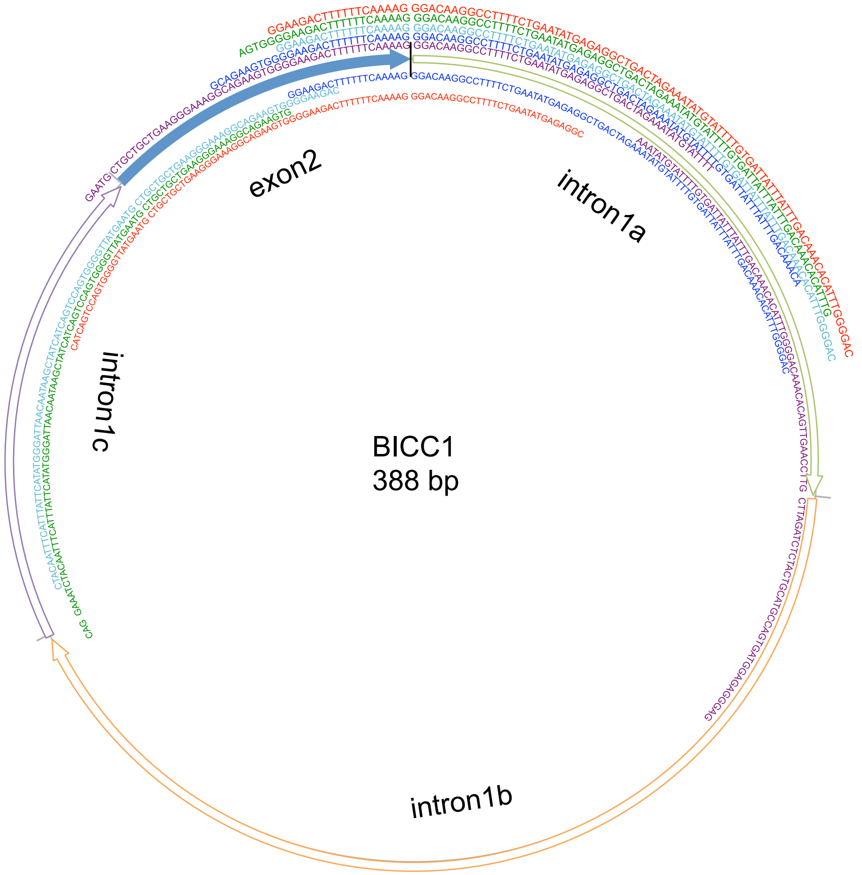

Suppl. Figure 7

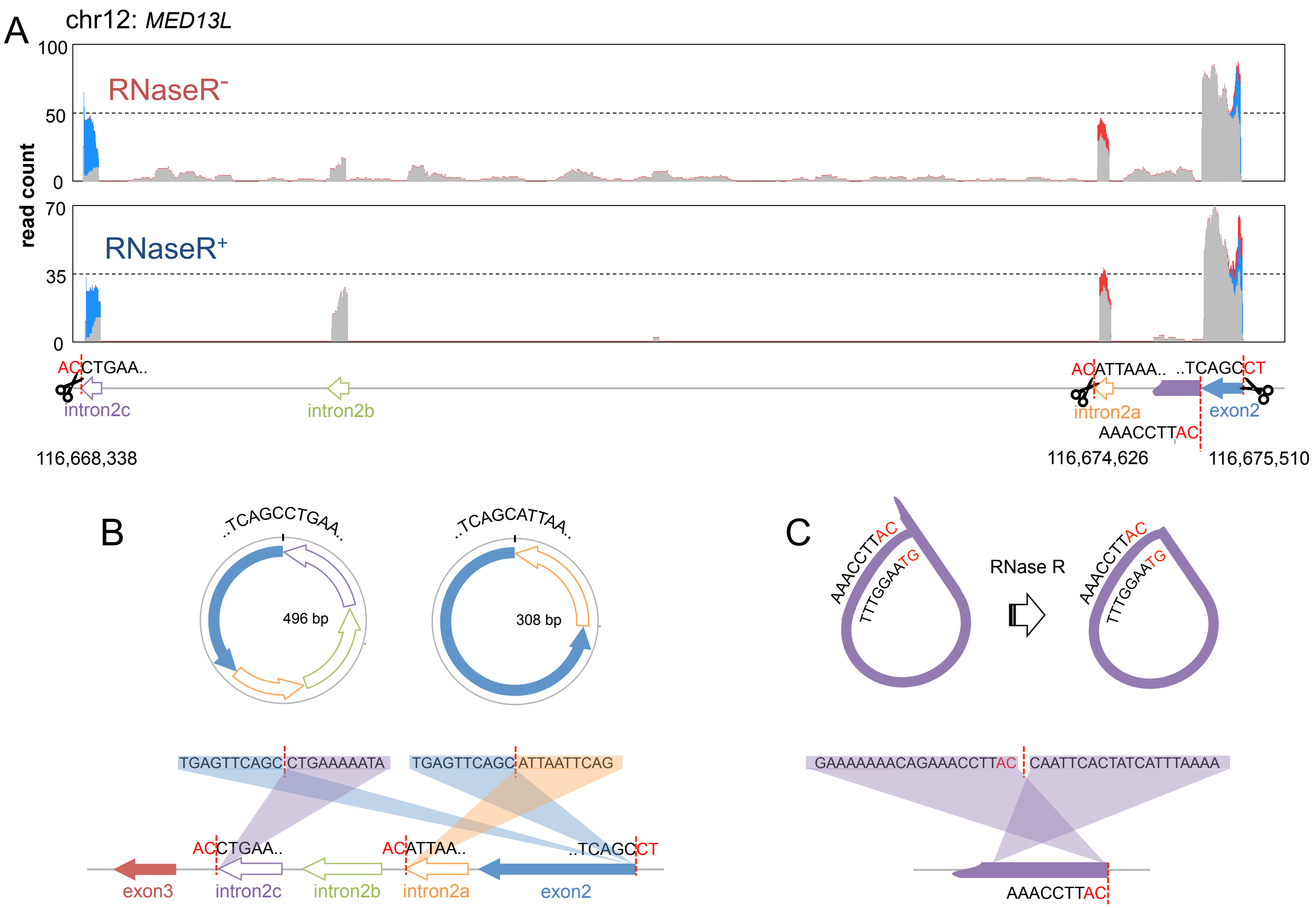

Suppl. Figure 8

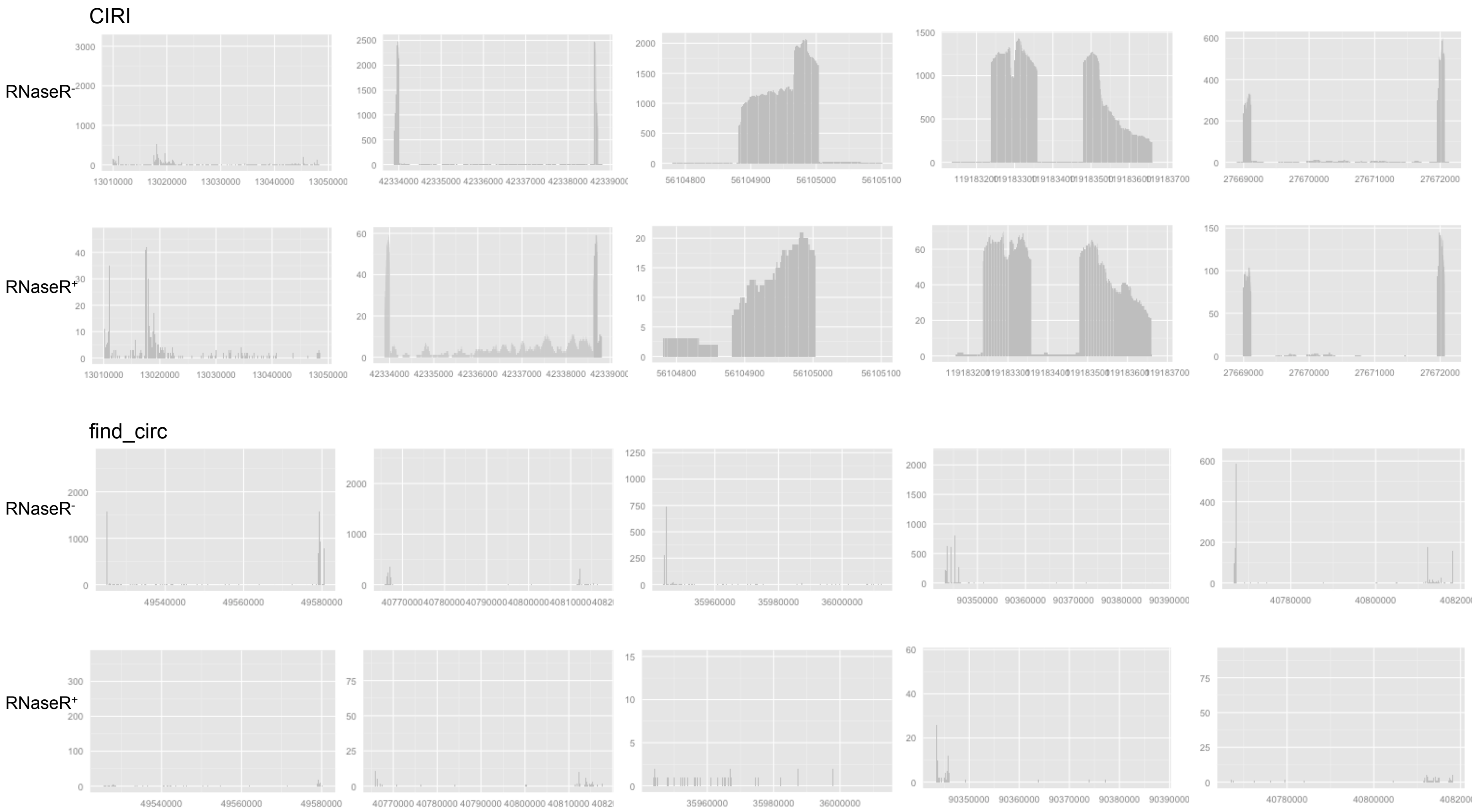

Suppl. Figure 9

A

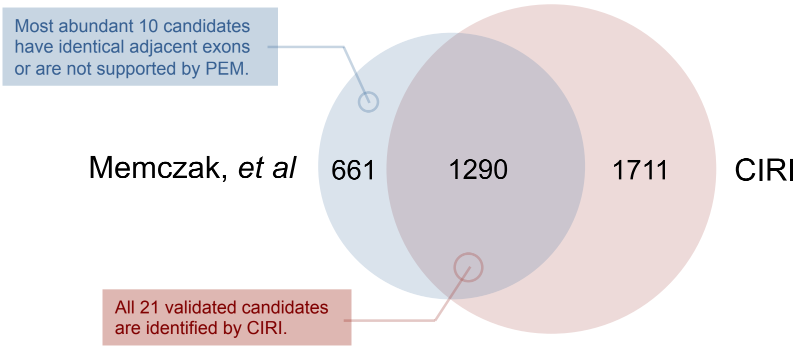

B

chr	Start	End	Name	Score	Unique	Questionable
**chr12**	49525080	49580616	hsa_circ_002179	2842	446	identical colinear exon
**chr14**	20811436	20811534	hsa_circ_002178	1400	214	not supported by PEM
**chr22**	29784412	29819177	hsa_circ_002177	226	53	identical colinear exon
**chr14**	106091232	106109862	hsa_circ_002174	106	32	identical colinear exon
**chr19**	45419446	45430290	hsa_circ_002169	83	21	identical colinear exon
**chr14**	20811404	20811554	hsa_circ_000167	80	25	not supported by PEM
**chr7**	141759655	141785761	hsa_circ_002166	75	31	identical colinear exon
**chr6**	32489734	32551938	hsa_circ_002165	74	19	identical colinear exon
**chr14**	106208677	106236323	hsa_circ_002164	73	27	not supported by PEM
**chr2**	231223678	231307814	hsa_circ_002162	63	25	not supported by PEM

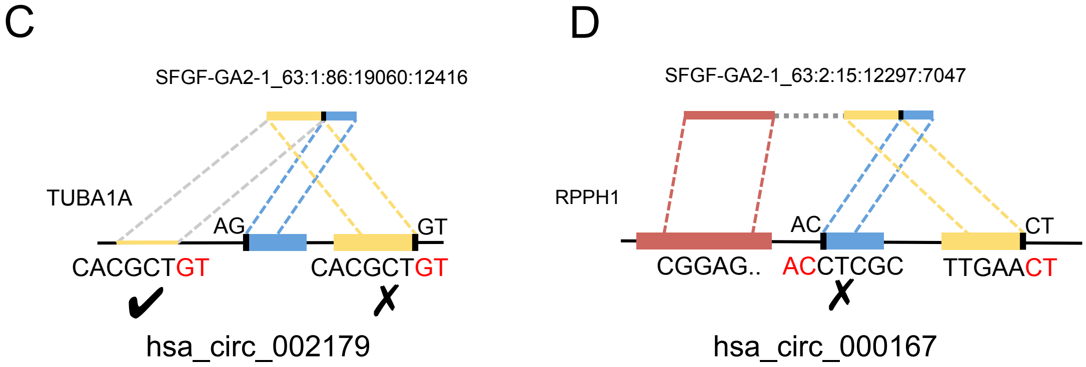

Suppl. Figure 10

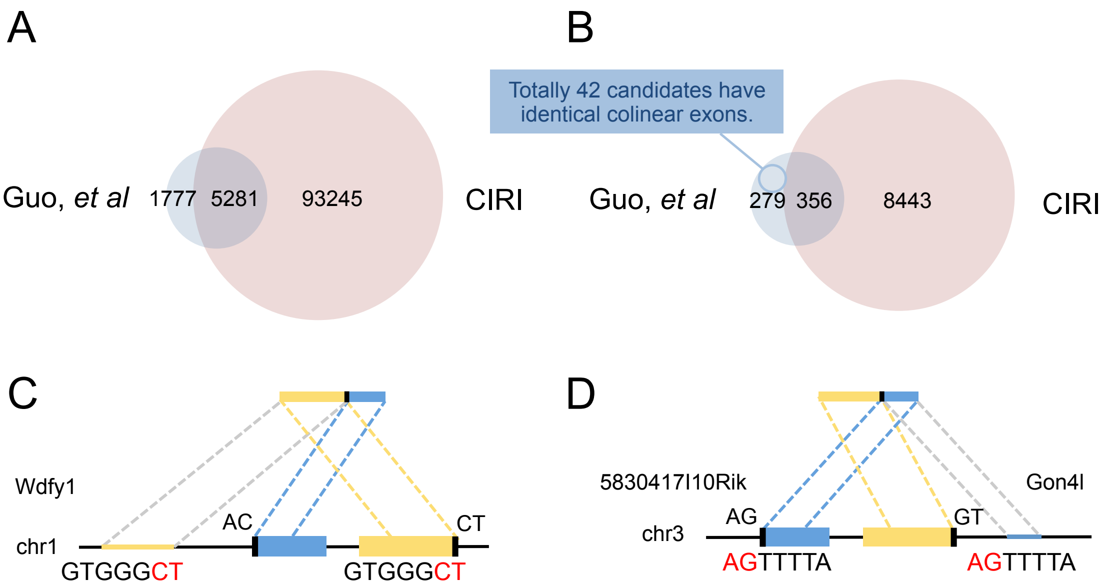

E

chr	Start	End	chr	Start	End
chr7	27844284	27862590	chr15	63849640	63915999
chr7	104315409	104348404	chr15	63869089	63902096
chr3	40839411	40887599	chr16	36184226	36221646
chr3	88815631	88892668	chr9	20441327	20473110
chr3	88825388	88899077	chr18	20279651	20336039
chr3	94665981	94764553	chr6	40724568	40750981
chr3	144759169	144817297	chr6	124516709	124573405
chr10	39894013	39971154	chr6	124519349	124561668
chr12	55180249	55252860	chr6	124537360	124631219
chr12	72628062	72657435	chr6	124542265	124636169
chr1	79722213	79726719	chr6	128931668	128953126
chr1	93835285	93909291	chr6	128931669	128944342
chr1	130422863	130462008	chrX	86356442	86388124
chr11	53873667	53956096	chrX	135310096	135380112
chr11	83212542	83243997	chrX	135796946	135839263
chr11	121907948	121944141	chrX	161223154	161224544
chr5	121604251	121622823	chr17	14986716	15048657
chr5	121605540	121626223	chr17	14987592	15018201
chr5	134360953	134362548	chr17	14988387	15044490
chr15	63818206	63880797	chr17	14999141	15027317
chr15	63847947	63880797	chr17	15021913	15051640

Suppl. Figure 11

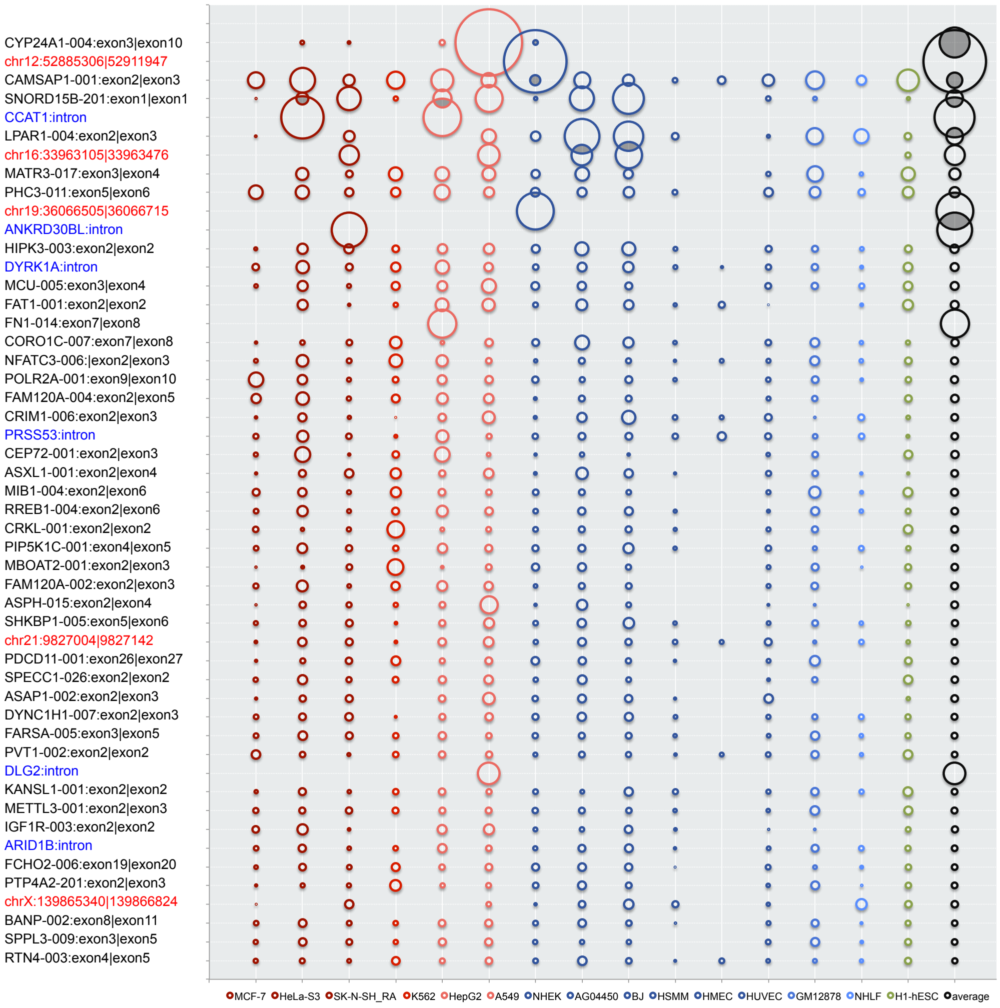

Suppl. Figure 12

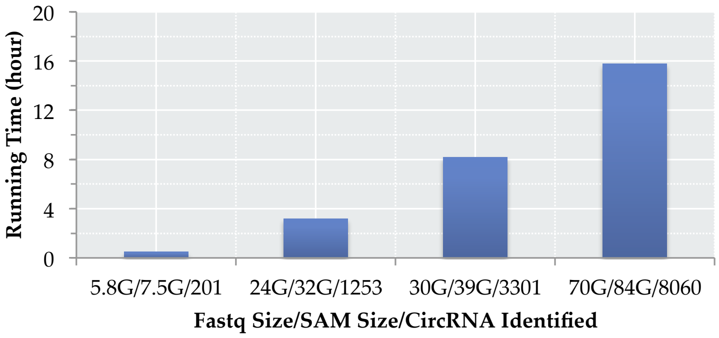

Suppl. Figure 13

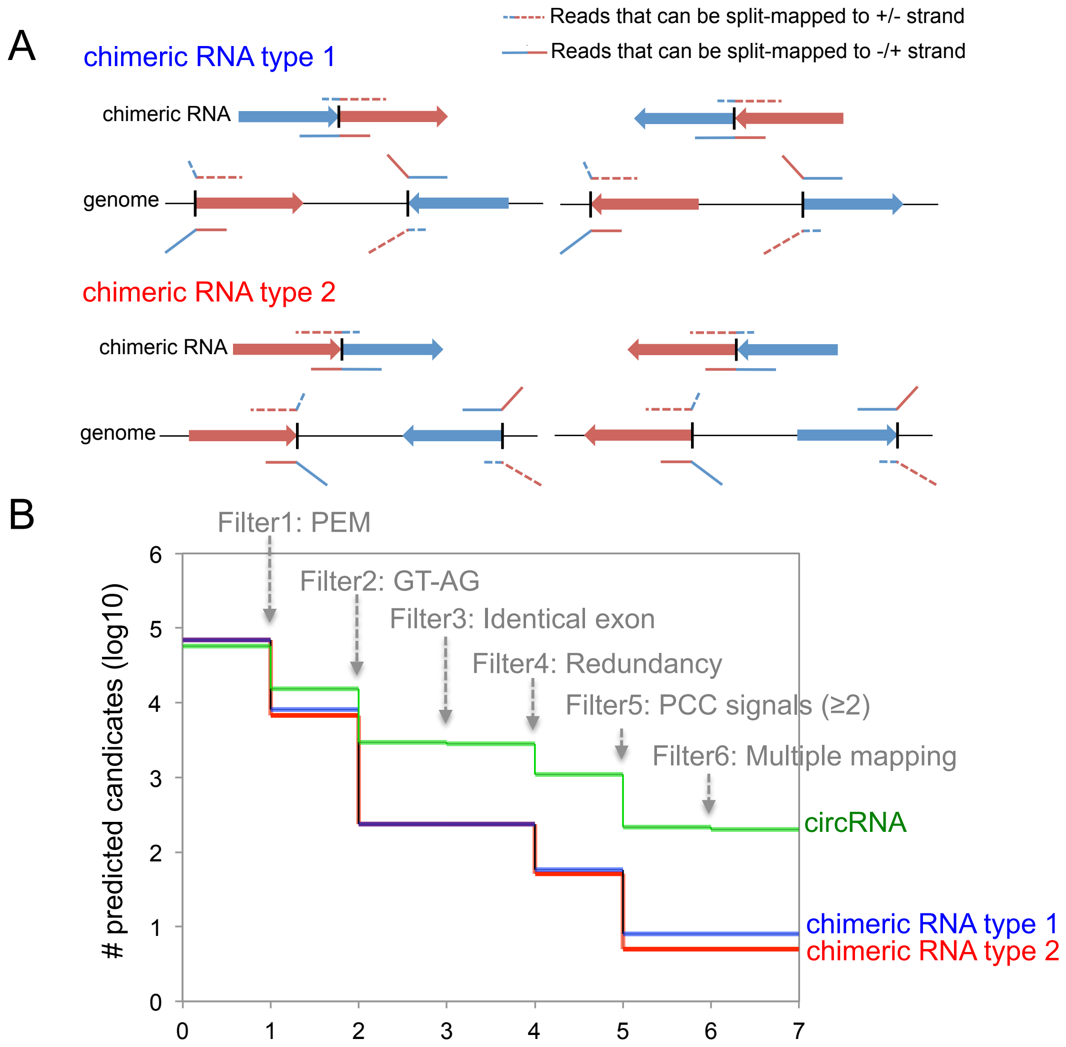

Suppl. Figure 14
